# Compatible Solute Synthesis and Import by the Moderate Halophile *Spiribacter salinus*: Physiology and Genomics

**DOI:** 10.3389/fmicb.2018.00108

**Published:** 2018-02-15

**Authors:** María J. León, Tamara Hoffmann, Cristina Sánchez-Porro, Johann Heider, Antonio Ventosa, Erhard Bremer

**Affiliations:** ^1^Department of Microbiology and Parasitology, Faculty of Pharmacy, University of Seville, Seville, Spain; ^2^Laboratory for Microbiology, Department of Biology, Philipps University of Marburg, Marburg, Germany; ^3^LOEWE-Center for Synthetic Microbiology, Philipps University of Marburg, Marburg, Germany

**Keywords:** *Spiribacter*, halophiles, hypersaline environments, osmoadaptation, ectoine, trehalose, glycine betaine, arsenobetaine

## Abstract

Members of the genus *Spiribacter* are found worldwide and are abundant in ecosystems possessing intermediate salinities between seawater and saturated salt concentrations. *Spiribacter salinus* M19-40 is the type species of this genus and its first cultivated representative. In the habitats of *S. salinus* M19-40, high salinity is a key determinant for growth and we therefore focused on the cellular adjustment strategy to this persistent environmental challenge. We coupled these experimental studies to the *in silico* mining of the genome sequence of this moderate halophile with respect to systems allowing this bacterium to control its potassium and sodium pools, and its ability to import and synthesize compatible solutes. *S. salinus* M19-40 produces enhanced levels of the compatible solute ectoine, both under optimal and growth-challenging salt concentrations, but the genes encoding the corresponding biosynthetic enzymes are not organized in a canonical *ectABC* operon. Instead, they are scrambled (*ectAC*; *ectB*) and are physically separated from each other on the *S. salinus* M19-40 genome. Genomes of many phylogenetically related bacteria also exhibit a non-canonical organization of the *ect* genes. *S. salinus* M19-40 also synthesizes trehalose, but this compatible solute seems to make only a minor contribution to the cytoplasmic solute pool under osmotic stress conditions. However, its cellular levels increase substantially in stationary phase cells grown under optimal salt concentrations. *In silico* genome mining revealed that *S. salinus* M19-40 possesses different types of uptake systems for compatible solutes. Among the set of compatible solutes tested in an osmostress protection growth assay, glycine betaine and arsenobetaine were the most effective. Transport studies with radiolabeled glycine betaine showed that *S. salinus* M19-40 increases the pool size of this osmolyte in a fashion that is sensitively tied to the prevalent salinity of the growth medium. It was amassed in salt-stressed cells in unmodified form and suppressed the synthesis of ectoine. In conclusion, the data presented here allow us to derive a genome-scale picture of the cellular adjustment strategy of a species that represents an environmentally abundant group of ecophysiologically important halophilic microorganisms.

## Introduction

The earth possesses widely distributed hypersaline ecosystems in which the main life-limiting factor is their high salt concentration (Ventosa et al., 1998; Grant, 2004). Most microbiological studies of these extreme ecosystems have been carried out on aquatic habitats, such as saline lakes and marine salterns. Salterns constitute excellent models for the study of the diversity and ecology of microorganisms that either strive or struggle under high-saline growth conditions (Ventosa et al., 2014, 2015).

Bacteria belonging to the genera *Halomonas, Chromohalobacter*, or *Marinobacter* can easily be isolated from such hypersaline environments and can readily be obtained in pure culture. Despite this, many of these isolates are now recognized as minor inhabitants of these ecosystems (Ghai et al., 2011; Fernandez et al., 2014a). In contrast, the isolation of the most abundant types of microorganisms from hypersaline habitats is challenging (Ventosa et al., 2014), because their cultivation for laboratory studies is difficult and tedious. As a consequence, the physiology of these microorganisms is typically assessed indirectly through metagenomic approaches (Ventosa et al., 2015), or, at best, studied in a few isolated species serving as proxy (Grote et al., 2012; Swan et al., 2013; Carini et al., 2014; Suh et al., 2015).

Members of the genus *Spiribacter* are abundant but elusive inhabitants of hypersaline ecosystems. The currently known species of the genus *Spiribacter, Spiribacter salinus* M19-40 (León et al., 2014), *Spiribacter curvatus* UAH-SP71 (León et al., 2015), *Spiribacter roseus* SSL50 (León et al., 2016), and *Spiribacter aquaticus* SP30 (León et al., 2017), have all been described as moderate halophiles. Depending on the particular species of these Gram-negative bacteria, members of the genus *Spiribacter* can grow in laboratory media with salinities ranging between 7.5 and 25% (w/v) and are worldwide distributed, as inferred from metagenomic studies of saltern ponds from Santa Pola (on the East coast of Spain), Isla Cristina (Southwest of Spain), and San Diego (CA, United States) (Lopez-Perez et al., 2013; Fernandez et al., 2014b; León et al., 2014, 2015; Ventosa et al., 2014). The complete genome sequence of the founding member of the genus *Spiribacter, S. salinus* M19-40, has recently been reported (León et al., 2013; Lopez-Perez et al., 2013). It is small in size (about 1.74 Mb) and provides the blueprint for a rather simple metabolic set-up (León et al., 2013; Lopez-Perez et al., 2013).

When one considers the lifestyle of halotolerant or halophilic microorganisms (Ventosa et al., 1998; Oren, 2013), it is important to recall that bacterial cells cannot actively pump water across their cytoplasmic membrane in a directed fashion. As a consequence, microorganisms have to compensate indirectly for water influxes or effluxes instigated by fluctuations in the external salinity in order to balance the magnitude of the vital osmotic gradient across their cytoplasmic membrane, and hence preserve vital turgor (Csonka, 1989; Kempf and Bremer, 1998; Bremer and Krämer, 2000; Roesser and Müller, 2001; Wood, 2011). They accomplish this by accumulating ions and organic osmolytes to raise the osmotic potential of the cytoplasm under hyperosmotic conditions to avoid dehydration and collapse of turgor (Kempf and Bremer, 1998), and they rapidly jettison these compounds through the transient opening of mechanosensitive channels when they are suddenly exposed to hypoosmotic circumstances to avoid an undue rise in turgor and eventual cell rupture (Booth, 2014).

The strategies used by microorganisms for their cellular adjustment to high salinity environments can be classified according to the *salt in* and *salt out* schemes (Galinski and Trüper, 1994; Kempf and Bremer, 1998; Wood et al., 2001), physiological osmostress responses that are not necessarily mutually exclusive (Deole et al., 2013; Oren, 2013; Youssef et al., 2014). Microorganisms that use the *salt in* strategy persistently accumulate large concentrations of K^+^ and Cl^-^ ions via transport, an energetically favorable process (Oren, 2011). However, the resulting high ionic strength of the cytoplasm required, on an evolutionary time scale, compensatory changes in practically the entire proteome in order to keep proteins soluble and to ensure the functionality of key cellular activities (Oren, 2013; Saum et al., 2013; Talon et al., 2014; Warden et al., 2015). This has left an acid signature on the proteome of those microorganisms that employ the *salt in* strategy and typically confine these bacteria and archaea to high salt habitats where the external salinity does not fluctuate frequently.

Although energetically more expensive (Oren, 2011), the *salt out* strategy is a physiological flexible osmostress adjustment response as it allows microorganisms to grow over a wide range of external salinities. In its core, it comprises the amassing of a selected class of organic osmolytes, the so-called compatible solutes, and the avoidance of a long-lasting large-scale accumulation of ions (Galinski and Trüper, 1994; da Costa et al., 1998; Kempf and Bremer, 1998; Bremer and Krämer, 2000; Roesser and Müller, 2001; Wood et al., 2001). The physico-chemical attributes of compatible solutes and their high water-solubility make them compliant with the proper folding of proteins and the biochemical functioning of key cellular processes (Bolen and Baskakov, 2001; Ignatova and Gierasch, 2006; Street et al., 2006; Zaccai et al., 2016; Stadmiller et al., 2017). Hence, there was no need to evolutionarily adjust the composition of the proteome to a high ionic strength cytoplasm through the acidification of protein surfaces. Compatible solutes can be accumulated to exceedingly high intracellular levels, and the external salinity dictates their cytoplasmic pool size (Kuhlmann A.U. et al., 2008; Brill et al., 2011; Hoffmann et al., 2013). Important representatives of compatible solutes used by both marine and terrestrial members of the bacteria are trehalose, proline, glycine betaine, ectoine/5-hydroxyectoine, glucosylglycerol, and dimethylsulfoniopropionate (DMSP) (Csonka, 1989; da Costa et al., 1998; Kempf and Bremer, 1998; Roesser and Müller, 2001; Klähn and Hagemann, 2011).

Once accumulated by microorganisms under high salinity growth conditions as stress protectants, compatible solutes are discharged into the environment upon osmotic downshifts through the transient opening of mechanosensitive channels or through cell lysis (Welsh, 2000; Booth, 2014). The released organic osmolytes are valuable environmental resources in a given ecosystem since they can be scavenged by other microorganisms via high-affinity transport systems either for their re-use as osmostress protectants (Kempf and Bremer, 1998; Bremer and Krämer, 2000; Wood et al., 2001) or for catabolic purposes (Welsh, 2000; Schwibbert et al., 2011; Schulz et al., 2017a,b).

Members of the genus *Spiribacter* belong to the family *Ectothiorhodospiraceae* within the *Gammaproteobacteria*. They represent the first ecologically defined moderate halophiles and are environmentally abundant inhabitants in various hypersaline ecosystems (Lopez-Perez et al., 2013; Fernandez et al., 2014b; León et al., 2014, 2015, 2016, 2017). Therefore, the type species of this genus, *S. salinus* M19-40 (León et al., 2014), can be considered as an interesting model organism for studies aiming at understanding how moderate halophiles cope with increased salinity, a trait critical for growth in and lasting colonization of the ecosystem they populate. Here, we have combined physiological approaches and *in silico* genome mining to derive a comprehensive picture of the salt-stress adjustment systems that allows *S. salinus* M19-40 to be an ecophysiological successful bacterium in hypersaline environments.

## Materials and Methods

### Chemicals

Glycine betaine, γ-butyrobetaine, and carnitine were purchased from Sigma-Aldrich (Steinheim, Germany). Arsenobetaine was obtained from Argus Chemical (Verino, Italy). Crotonobetaine was generously provided by J. Brass (Lonza AG, Visp, Switzerland). DMSP was from a laboratory stock and was custom synthesized as described (Nau-Wagner et al., 1999; Broy et al., 2015). Homobetaine and choline-*O*-sulfate were kind gifts from G. Nau-Wagner (University of Marburg, Germany), and ectoine and 5-hydroxyectoine were generously provided by bitop AG (Witten, Germany). Radiolabeled [1-^14^C]glycine betaine (55 mCi mmol^-1^) was purchased from American Radiolabeled Chemicals (St. Louis, MO, United States).

### Bacterial Strains, Growth Media, and Culture Conditions

All experiments were conducted with the type species of the genus *Spiribacter, S. salinus* strain M19-40 (León et al., 2014). It is available from several culture collections under accession numbers CECT 8282^T^ (Spanish Type Culture Collection), IBRC-M 10768^T^ (Iranian Biological Resource Center), and LMG 27464^T^ (Belgian Coordinated Collection of Microorganisms).

Cultures of *S. salinus* M19-40 (40-ml medium in a 100-ml Erlenmeyer flask) were inoculated from exponentially growing pre-cultures and incubated at 37°C in a water bath or aerial shaker set to 180 rpm. Their growth was monitored photometrically as optical density at 600 nm (OD_600_). *S. salinus* M19-40 was routinely cultivated in a modified SMM medium (León et al., 2014) which has the following composition (w/v): 1.95% MgCl_2_.6H_2_O, 3.05% MgSO_4_.7H_2_O, 0.05% CaCl_2_, 0.3% KCl, 0.01% NaHCO_3_, 0.035% NaBr, 0.5% casein digest, and 0.11% sodium pyruvate. The pH of the growth medium was adjusted to 7.5 with 4 M KOH. The osmolarity of the growth medium was adjusted by adding NaCl from a 5-M stock solution as required by the set-up of the individual experiments. Solutions of the compatible solutes used for osmostress protection assays were sterilized by filtration and added to media from 100 mM stock solutions to a final concentration of 1 mM.

### Solute Extraction for the Quantification of Ectoine and Trehalose

Cultures of *S. salinus* M19-40 were grown at 37°C in SMM containing various amounts of NaCl until they reached the exponential phase (depending on the NaCl concentration of the medium the cultures had OD_600_ values that varied between 0.04 and 0.2). Twenty milliliter aliquots were withdrawn from the cultures, the cells were harvested by centrifugation and the pellets were solvent extracted in 1 ml ethanol [20% (v/v)] for 1 h at room temperature. After centrifugation (16,000 × *g* for 30 min at 4°C), the cell free extracts containing compatible solutes and other soluble material were recovered and dried at 60°C for 24 h. The dried compounds were re-suspended in 120 μl H_2_O and the samples were then centrifuged (16,000 × *g* for 30 min at 4°C) to remove water-insoluble particles. The supernatants of these samples were then subsequently used for quantification of ectoine and trehalose.

### Quantification of Ectoine

Ectoine was detected by high-performance liquid chromatography (HPLC) analysis using an Agilent 1260 Infinity LC system (Agilent, Waldbronn, Germany) and a GROM-SIL Amino 1PR column (GROM, Rottenburg-Hailfingen, Germany) essentially as described (Kuhlmann and Bremer, 2002) with the exception that a 1260 Infinity Diode Array Detector (DAD) (Agilent) was employed, instead of the previously used UV/Vis system. The ectoine content of samples was quantified using the OpenLAB software suite (Agilent). The individual values are given as intracellular ectoine concentration (μM) in cultures of *S. salinus* M19-40 that correspond to an OD_600_ of 1.

### Quantification of Trehalose

Trehalose was detected via a coupled enzyme reaction assay using the “Trehalose Assay Kit” purchased from Megazyme (Wicklow, Ireland). In this assay, the trehalose concentration of the samples is quantified by the photometrical determination of NADPH at a wavelength of 340 nm after the trehalase is stoichiometrically converted into gluconate-6-phosphate and NADPH by the activity of the enzymes trehalose, hexokinase, and glucose-6-phosphate dehydrogenase. To avoid background reactions of contaminating sugars, an alkaline borohydride reduction of our samples was performed. The alkaline borohydride reduction and the enzymatic reaction were performed as detailed in the manual of the Megazyme trehalose assay kit with the exception that we calculated the trehalose concentrations of the samples using a standard curve derived from the parallel measurement of trehalose standards with concentrations ranging between 0 and 1000 μM. The individual values are given as intracellular trehalose concentration (μM) in a *S. salinus* M19-40 culture that corresponds to an OD_600_ value of 1.

### Determination of Glycine Betaine Pools

Intracellular glycine betaine pools were analyzed in *S. salinus* M19-40 cells that were cultivated in media with various NaCl concentrations in the presence of 1 mM unlabeled glycine betaine that was spiked with 0.64 μM radiolabeled [1-^14^C]glycine betaine. After the cultures reached late exponential growth phase, 3-ml aliquots were filtrated onto membrane filters (Whatman ME25 0.45 μM, GE Healthcare, Munich, Germany). The cells on the filters were washed with isotonic growth medium, and the radioactivity of the cells measured by scintillation counting was used to calculate the intracellular glycine betaine concentrations as described previously (Holtmann et al., 2003). The individual values are given as intracellular glycine betaine concentration (μM) in cells that correspond to an OD_600_ value of 1. To further prove that the cells accumulate glycine betaine, and no metabolic products of it, we separated the intracellular solute pool of the cells by thin-layer chromatography (TLC). One milliliter samples taken from the cultures used to assess the intracellular glycine betaine pools were harvested by centrifugation (16,000 × *g* for 30 min at 4°C). Lysis of the cells, spotting of the intracellular solutes on a TLC plate (Silica Gel G; Macherey-Nagel, Düren, Germany) and separation by TLC was performed as described previously (Boch et al., 1994). Radiolabeled compounds were visualized using a Storm 840 phosphorimager (GE Healthcare, Munich, Germany), and the strength of the derived signals was quantified with the ImageQuant^TM^ software package (GE Healthcare, Munich, Germany); these values were normalized to an OD_600_ of 1 of the *S. salinus* M19-40 cultures.

### Bioinformatic Analysis of the Osmostress Response Systems of *S. salinus* M19-40

The amino acid sequence of the EctC protein of *S. salinus* M19-40 (SPISAL_06145; NCBI genome accession number CP005963), the diagnostic enzyme of the ectoine biosynthetic route (Ono et al., 1999; Widderich et al., 2014, 2016), was used in a BLAST search analysis at the Integrated Microbial Genomes & Microbiomes (IMG/M) system of the Department of Energy DOI^1^ (Nordberg et al., 2013). The genomes of the 35 microorganisms exhibiting the most closely related EctC amino acid sequences to the *S. salinus* M19-40 EctC protein were further analyzed using the toolbox provided by the web-server of the IMG/M database. A phylogenetic tree was built using the IMG/M “distance tree” tool which calculates phylogenetic distances and creates a tree using the alignment of 16S rRNA gene sequences based on the SILVA database^2^ and dnadist and neighbor tools from the PHYLIP package^3^. For cases where the exact gene sequence cannot be found in the SILVA database, the sequence with the highest similarity is automatically selected. Subsequently, the *ectC* gene neighborhoods of the studied 35 microorganisms were analyzed to assess the occurrence of further ectoine related genes. This assessment was extended via subsequent BLAST searches using the protein sequences of the ectoine hydroxylase (EctD) (accession number CBV43892.1) and the EctB enzyme (accession number OBX36375) from *Halomonas elongata* (Schwibbert et al., 2011) as search queries. This identified corresponding EctD and EctB homologs from *S. salinus* M19-40 and from those 35 *S. salinus*-related species that contained EctC. The amino acid sequences of the putative ectoine hydroxylases were compared and analyzed for the presence of the EctD consensus sequence (Reuter et al., 2010; Widderich et al., 2014) and residues involved in substrate binding and catalysis of this enzyme (Höppner et al., 2014). The genome sequence of *S. salinus* M19-40 (León et al., 2013; Lopez-Perez et al., 2013) was also searched for various types of compatible solute synthesis, degradation, and import proteins using query sequences derived from various microorganisms that have been physiologically and genetically studied for these traits in the past. A summary of the templates used for this *in silico* mining of the *S. salinus* M19-40 genome and the corresponding E-values of the retrieved *S. salinus* M19-40 proteins are given in Supplementary Table 1.

### Generation of Protein Structure Models

Modeling of putative glycine betaine ligand-binding proteins from *S. salinus* M19-40 was conducted using the Swiss-Model protein structure prediction platform^4^ (Arnold et al., 2006). The resulting *in silico* structure models were compared with those of the crystal structures of corresponding extracellular solute receptor proteins from the *Escherichia coli* ProU and *Bacillus subtilis* OpuA ABC-transporters [Protein Database (PDB) accession codes for the ProX::glycine betaine and the OpuAC::glycine betaine complexes are 1R9L and 2B4L, respectively) (Schiefner et al., 2004; Horn et al., 2006). An *in silico* model of a predicted ectoine/5-hydroxyectoine ligand-binding protein (TeaA) of the tripartite ATP-independent periplasmic (TRAP) family transporter TeaABC (Grammann et al., 2002) from *S. salinus* M19-40 was compared with the crystal structure of the corresponding solute receptor protein from *H. elongata* (PDB accession code 2VPN) (Kuhlmann S.I. et al., 2008). Graphic representations of the various *in silico* generated models of glycine betaine and ectoine ligand-binding protein complexes from *S. salinus* M19-40 were prepared using the PyMOL software package^5^ (Delano, 2002).

## Results

### Growth Properties of *S. salinus* M19-40 in Response to Increases in Environmental Salinity

**FIGURE 1:**
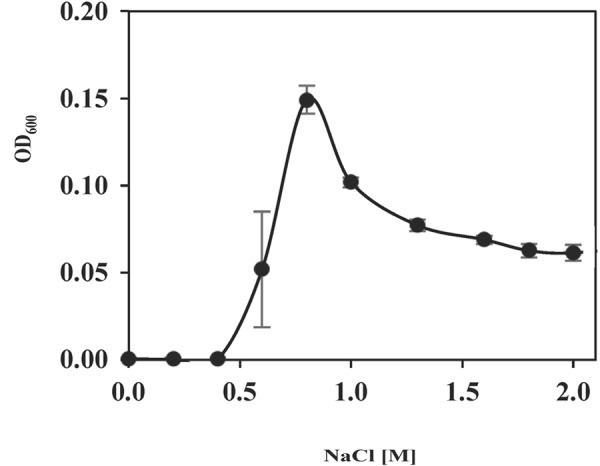
Growth of *S. salinus* M19-40 at increasing salinities. To determine the growth properties of *S. salinus* M19-40 in response to various concentrations of NaCl in the medium, the cells were cultured for 90 h in SMM at 37°C and their growth yields were determined by measuring the OD_600_. Given are the averaged values and standard deviations derived from three independently grown cultures.

To assess the amount of NaCl needed for its growth and the ability of *S. salinus* M19-40 to colonize hypersaline habitats, cells were propagated in SMM with increasing concentrations of NaCl (from 0 to 2.0 M NaCl). No growth was detectable up to a salinity of 0.4 M NaCl (**Figure 1**), a property expected for a moderate halophile (León et al., 2014; Ventosa et al., 2014, 2015). An increase in the salinity up to 0.8 M NaCl strongly stimulated growth, but further increases (from 1.0 to 2.0 M NaCl) successively impaired growth. Hence, *S. salinus* M19-40 not only depends on a considerable salt concentration for its growth but it can also cope with a broad spectrum of salinities (from 0.6 to 2.0 M NaCl). However, its growth is optimally poised for a rather narrow spectrum of salinities (**Figure 1**).

### Mining the Genome Sequence for Systems Mediating K^+^ and Na^+^ Homeostasis

**FIGURE 2:**
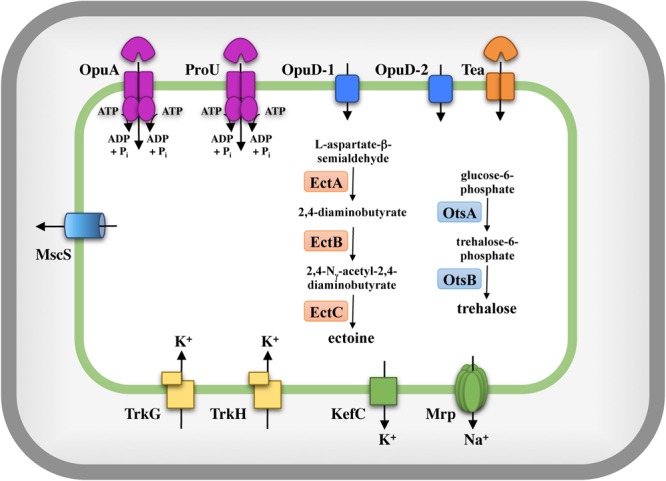
*In silico* model of the osmostress response systems of *S. salinus* M19-40. *In silico* analysis of the genome sequence of *S. salinus* M19-40 (León et al., 2013; Lopez-Perez et al., 2013) with respect to uptake- and release systems for ions and compatible solutes, compatible solute biosynthesis routes, and mechanosensitive channels, systems which are known from previous studies to be involved in osmostress adaptation in members of the bacteria. The depicted systems are based on bioinformatics data summarized in Supplementary Table 1.

The stress response systems of microorganisms which use the *salt out* adaptation strategy to high-salinity environments typically comprise three interwoven cellular adjustment processes: (i) the import of large quantities of potassium ions, (ii) the accumulation of compatible solutes either through synthesis or uptake, and (iii) the reduction of the ionic strength of the cytoplasm through the export of previously imported potassium ions (Csonka, 1989; Kempf and Bremer, 1998; Bremer and Krämer, 2000; Wood et al., 2001; Wood, 2011). Furthermore, the cell must ensure that the cellular levels of cytotoxic sodium ions are kept at a very low concentration (Gorecki et al., 2014). These processes have, for instance, been intensively studied in *E. coli, Salmonella typhimurium, B. subtilis, Corynebacterium glutamicum, H. elongata*, and *Chromohalobacter salexigens* (Csonka, 1989; Kempf and Bremer, 1998; Wood et al., 2001; Morbach and Krämer, 2002; Vargas et al., 2008; Schwibbert et al., 2011; Hoffmann and Bremer, 2016). We took advantage of the analysis of the osmostress response systems of these Gram-negative and Gram-positive microorganisms to derive a plausible *in silico* picture of the osmostress response system of *S. salinus* M19-40. The results of these efforts are summarized in **Figure 2** and are discussed in some detail below.

Fluxes and pools of potassium play key roles in the cellular response to both suddenly imposed and sustained osmotic and salt stress in many microorganisms (Dinnbier et al., 1988; Whatmore et al., 1990). *S. salinus* M19-40 possesses two copies of Trk-type potassium uptake systems (**Figure 2**); TrkG (SPISAL_06570 and SPISAL_06575) and TrkH (SPISAL_00315 and SPISAL_00310) (Diskowski et al., 2015). These types of active potassium importers possess a membrane-embedded K^+^-translocating subunit operating in conjunction with a peripheral membrane protein containing an RCK-domain (RCK: regulation of K^+^ channel), which forms a gating ring controlling the activity of the transporter (Diskowski et al., 2015; Szollosi et al., 2016). Genes for other types of well-studied K^+^ import systems, such as Kup, Kdp, and Ktr (Diskowski et al., 2015), or the potassium channel CglK from *C. glutamicum* (Ochrombel et al., 2011) are not found in the *S. salinus* M19-40 genome sequence.

Cellular potassium pools are tightly regulated not only through import but also through efflux. We detected a gene (SPISAL_01180) for a KefC-type potassium efflux system (Epstein, 2003) in the genome of *S. salinus* M19-40. Typically, maximal activity of KefC-type potassium efflux systems is attained through interactions with other types of proteins, such as KefF, KefG, or AmhM. No genes related to these latter three proteins were found in our database search of the *S. salinus* M19-40 genome sequence. We did not find any homologs to other potassium extrusion systems, such as of the *B. subtilis* YugO and KhtTU from *B. subtilis* (Fujisawa et al., 2007; Lundberg et al., 2013).

Further inspection of the *S. salinus* M19-40 genome sequence (León et al., 2013; Lopez-Perez et al., 2013) revealed the presence of genes for a multi-component Mrp sodium extrusion system (Swartz et al., 2005). Mrp-type sodium exporters are widely found in microorganisms and play key roles in monovalent inorganic cation homeostasis, pH regulation, and energy metabolism (Swartz et al., 2005). The activity of the Mrp system is very important for ensuring growth in high salinity environments; for instance, its mutational inactivation in *B. subtilis* leads to a pronounced sensitivity to NaCl (Gorecki et al., 2014). In *S. salinus* M19-40, the genes for the Mrp-system (SPISAL_06635 to SPISAL_06680) appear to be organized as an operon comprising ten genes (Supplementary Table 1). This is a somewhat unusual number of genes since typical Mrp system consists of six to seven subunits (Swartz et al., 2005) but recent genome surveys of many bacteria and archaea revealed *mrp* operons with diverse numbers of genes (Ito et al., 2017). An overview of the Mrp proteins from *S. salinus* M19-40, and their predicted function is given in Supplementary Table 1. Genome searches for the presence of the genes for other types of dedicated sodium extrusion systems such as NatAB, NhaC, and NhaK did not retrieve any hits in *S. salinus* M19-40.

### Mining the Genome Sequence for Compatible Solute Import Systems

Import of compatible solutes contributes greatly to the cellular defense of many microorganisms to the physiological challenges imposed by high salinity surroundings (Csonka, 1989; Kempf and Bremer, 1998; Bremer and Krämer, 2000; Roesser and Müller, 2001; Wood et al., 2001). We therefore searched the *S. salinus* M19-40 genome for various types of compatible solute transporters and found five distinct systems that can be related to known osmolyte uptake systems (**Figure 2**).

Two of these predicted osmolyte importers are single-component systems (OpuD-1 and OpuD-2) (SPISAL_05155 and SPISAL_05630, respectively) and these two proteins possess a high degree (41%) of amino acid sequence identity to each other. BLAST searches (Altschul et al., 1990) and amino acid comparison of the most closely related proteins demonstrated that the *S. salinus* M19-40 OpuD-1 and OpuD-2 proteins belong to the betaine–choline–carnitine transporter (BCCT) family (Kappes et al., 1996; Ziegler et al., 2010). Many members of this widespread transporter family mediate the uptake of various types of osmoprotectants, including proline, choline, glycine betaine, arsenobetaine, DMSP, and ectoine (Ziegler et al., 2010; Hoffmann et al., 2017). When the amino acid sequences of the OpuD-1 and OpuD-2 proteins were separately used for BLAST searches using the UniProt knowledgebase (Pundir et al., 2017), the list of the top 17–20 recovered hits was very similar and included functionally characterized members of the BCCT family (Ziegler et al., 2010), including BetS from *Rhizobium meliloti*, BetT-1 and BetT-2 from *Acinetobacter baylyi*, OpuD from *B. subtilis*, BetL from *Listeria monocytogenes*, EctP, BetP, and LcoP from *C. glutamicum*, BetT from *E. coli*, EctT from *Virgibacillus pantothenticus*, and several BCCT-system from *Vibrio parahaemolyticus* (Supplementary Figure 1).

The *S. salinus* M19-40 genome also encodes two ABC transporters that are in all likelihood involved in compatible solute uptake; the OpuA and ProU systems (SPISAL_06400 to SPISAL_06410, and SPISAL_05285 to SPISAL_05290, respectively) (**Figure 2**). The ProU transporter has been intensively studied both in *E. coli* and in *S. typhimurium* and is a binding-protein-dependent ABC-type uptake system (Lucht and Bremer, 1994). A crystal structure of the corresponding periplasmic ligand binding protein (ProX) of the *E. coli* ProU system in complex with glycine betaine has been determined (Schiefner et al., 2004). Our amino acid sequence comparison between the *E. coli* ProX and SPISAL_05285 proteins revealed a 31% sequence identity. Our modeling studies of the SPISAL_05285 protein that are based on the ProX::glycine betaine complex (Schiefner et al., 2004) showed that those residues coordinating the glycine betaine ligand within the active site are conserved (Supplementary Figure 4).

The second predicted ABC-type osmostress protectant uptake system of *S. salinus* M19-40 (SPISAL_06400 to SPISAL_06410) is related to the OpuA glycine betaine transporter from *B. subtilis* (Kempf and Bremer, 1995). The substrate-binding protein (OpuAC) from the Gram-positive bacterium *B. subtilis* is a lipoprotein tethered to the outer face of the cytoplasmic membrane, whereas the corresponding protein (SPISAL_06400) from the Gram-negative bacterium *S. salinus* M19-40 is a predicted periplasmic protein (León et al., 2013, 2014). In comparison with the OpuAC crystal structure (Horn et al., 2006), the two lobes of the SPISAL_06400 protein are inversed, a phenomenon that has previously been observed for a substantial group of OpuAC-related proteins (Smits et al., 2008; Wolters et al., 2010). When this is taken into account and an OpuAC-based homology model of the SPISAL_06400 protein is constructed (Supplementary Figure 5), we found that the aromatic cage coordinating the positively charged trimethylammonium head-group of glycine betaine within the binding site of the *B. subtilis* OpuAC protein via cation-π interactions is strictly conserved in the SPISAL_06400 protein (Supplementary Figure 5B). It is worth noting in this context that the *E. coli* ProU and the *B. subtilis* OpuA systems provide the cells with uptake capacity for various types of osmostress protectants (Haardt et al., 1995; Hoffmann and Bremer, 2011).

Finally, we also found genes for a TeaABC-type TRAP transporter (SPISAL_01885 to SPISAL_01895) (**Figure 2**) in the genome of *S. salinus* M19-40. The prototype of this transporter was characterized in *H. elongata* (Grammann et al., 2002) where it functions as an import system for ectoine and 5-hydroxyectoine when they are taken up as osmostress protectants. The TeaABC transporter also serves as a recycling system for newly synthesized ectoines that either leak or are actively exported from osmotically stressed *H. elongata* cells, a function that becomes apparent in a *H. elongata* mutant with a genetically inactivated TeaABC system (Grammann et al., 2002). The activity of many TRAP transporters is energized by sodium gradients, and consequently, these types of binding-protein-dependent import systems are particularly prevalent in microorganisms living either in marine or in highly saline habitats (Mulligan et al., 2011).

The ligand-binding protein (SPISAL_01895) of the TeaABC system of *S. salinus* M19-40 exhibits an amino acid sequence identity of 78 and 57% to the corresponding proteins (TeaA and UehA) from the halophile *H. elongata* and the marine bacterium *Ruegeria pomeroyi* DSS-3, respectively (Grammann et al., 2002; Lecher et al., 2009). This latter protein is part of a substrate-inducible ectoine/5-hydroxyectoine import system (UehABC) used by *R. pomeroyi* DSS-3 to scavenge these compounds as nutrients from environmental sources (Schulz et al., 2017a,b). Despite that the TeaABC and UehABC transporters are used for different physiological purposes, the crystal structures of the 5-hydroxyectoine ligand-binding proteins TeaA and UehA are closely related to each other and their binding sites are virtually superimposable (Kuhlmann S.I. et al., 2008; Lecher et al., 2009). Modeling of the SPISAL_01895 binding protein based on the *H. elongata* TeaA crystal structure (Kuhlmann S.I. et al., 2008) revealed a putative ligand-binding site in which the residues mediating binding of ectoines are strictly conserved (Supplementary Figure 6). Hence, the TeaABC system of *S. salinus* M19-40 seems to be an ectoine/5-hydroxyectoine-specific uptake system.

A BLAST search using either the TeaA or UehA proteins as the search query identified the presence of an additional TRAP transporter in *S. salinus* M19-40 (SPISAL_04635 to SPISAL_04645). However, a closer inspection of the corresponding ligand-binding protein (SPISAL_04635) revealed that key determinants for ectoine/5-hydroxyectoine binding by the solute receptors TeaA (Kuhlmann S.I. et al., 2008) and UehA (Lecher et al., 2009) are missing, suggesting that this particular TRAP transporter is not involved in capturing ectoines from external sources.

Sudden osmotic down-shifts require a very rapid adjustment cellular response since the ensuing influx of water can potentially drive up turgor to values that cannot be restrained by the peptidoglycan sacculus (Booth, 2014). Many microorganisms therefore possess safety valves, the mechanosensitive channels, that open transiently to release ions and organic compounds in order to reduce the osmotic potential of the cytoplasm to curb water influx (Booth, 2014). *S. salinus* M19-40 possesses a gene (SPISAL_03885) for a mechanosensitive channel of small conductance (MscS) (**Figure 2**) but it lacks a gene for a channel of large conductance (MscL).

Many microorganisms possess AqpZ-type aquaporins, channels that can mediate accelerated water fluxes (Hedfalk et al., 2006). However, the physiological role of these types of aquaporins for osmotic adjustment of bacteria is not entirely clear (Tanghe et al., 2006) and *aqpZ*-type genes are frequently not present in many microorganisms (Calamita, 2000). The genome sequence of *S. salinus* strain M19-40 lacks an *aqpZ*-type gene.

### Mining the Genome Sequence for Compatible Solute Synthesis Pathways

In addition to compatible solute import, the synthesis of these types of physiologically compliant organic osmolytes is a common strategy to adjust the cytoplasmic solute pool under unfavorable growth conditions (Csonka, 1989; da Costa et al., 1998; Kempf and Bremer, 1998; Bremer and Krämer, 2000; Roesser and Müller, 2001; Wood et al., 2001). We found genes for the osmostress-responsive synthesis of trehalose and ectoine in the genome sequence of *S. salinus* M19-40.

Trehalose is a non-reducing disaccharide synthesized widely used as compatible solute by microorganisms. It serves not only as an osmostress protectant, but also confers protection against low- and high-temperature stress and preserves the functionality of macromolecules and cells under desiccation stress (Strom and Kaasen, 1993). Trehalose can be produced in microorganisms through various biosynthetic pathways, the most widely distributed is the route catalyzed by OtsAB enzymes (Avonce et al., 2006). In this biosynthetic pathway, glucose-6-phosphate is used as the precursor and is converted into trehalose-6-phosphate by the α, α-trehalose-phosphate synthase (OtsA). This intermediate is then dephosphorylated by the trehalose-6-phosphate phosphatase (OtsB) to form free trehalose (Strom and Kaasen, 1993). *S. salinus* M19-40 possesses the corresponding genes (*otsB and otsA*; SPISAL_07860 and SPISAL_07870; **Figure 2**). In *E. coli*, these genes are transcribed as an osmotically inducible operon (Hengge-Aronis et al., 1991; Strom and Kaasen, 1993), while in *S. salinus* M19-40 they are separated by a gene (SPISAL_07865) that is predicted to code for a member of the glycoside hydrolase 15 family. This protein family comprises enzymes that can hydrolyze the glycosidic bond between two or more carbohydrates (Bourne and Henrissat, 2001). The precursor for trehalose synthesis, glucose-6-phosphate, is frequently generated through import of glucose via a PTS-transporter (Boos et al., 1990). In our database searches of the *S. salinus* M19-40 genome, we did not find evidence for glucose-, maltose-, or trehalose-specific components (protein EII) for PTS systems for these sugars. However, as already reported for *C. salexigens* (Vargas et al., 2008), a glucokinase (SPISAL_03590) (Supplementary Table 1) can be found in *S. salinus* M19-40 that potentially could provide the precursor for trehalose synthesis.

A previous inspection of the *S. salinus* M19-40 genome sequence indicated that this bacterium should be able to synthesize the compatible solute ectoine (Lopez-Perez et al., 2013). Synthesis of ectoine proceeds from L-aspartate-β-semialdehyde and comprises three enzymatic steps that are catalyzed by L-2,4-diaminobutyrate transaminase (EctB), 2,4-diaminobutyrate acetyltransferase (EctA), and ectoine synthase (EctC) to yield the cyclic ectoine molecule [(4S)-2-methyl-1,4,5,6-tetrahydropyrimidine-4-carboxylic acid] (Louis and Galinski, 1997; Ono et al., 1999). Some microorganisms are also able to synthesize a hydroxylated derivative of ectoine, the effective cytoprotectant 5-hydroxyectoine, in a stereo- and region-specific reaction that is catalyzed by the ectoine hydroxylase (EctD) (Prabhu et al., 2004; Garcia-Estepa et al., 2006; Bursy et al., 2007; Höppner et al., 2014).

**FIGURE 3:**
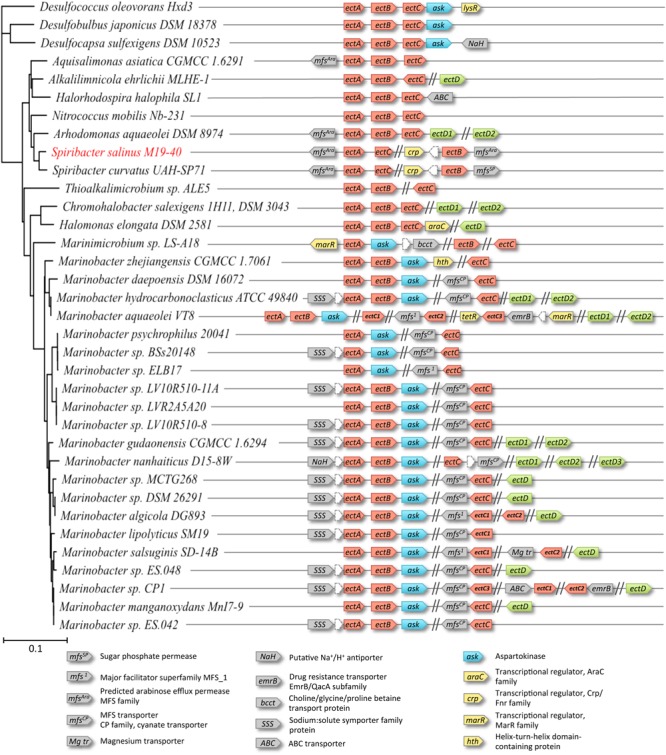
Genetic organization of the ectoine biosynthetic clusters in *S. salinus* M19-40 and phylogenetically related microorganisms. Genes whose gene products might be involved in ectoine (red), or hydroxyectoine (green) biosynthesis were searched by BLAST analysis in the genomes of *S. salinus* M19-40 and taxonomically related organisms. The 16S-rRNA based phylogenetic relation of the organisms and the genetic organization of the *ect*-genes and their genomic neighborhoods are shown. The annotation and distribution of an aspartokinase (*ask*) associated with ectoine synthesis (blue), putative transporter (gray), or regulator proteins (yellow) encoded in the neighborhood of the *ect* genes are highlighted.

The structural genes for the ectoine biosynthetic enzymes are typically organized in an operon (*ectABC*; Louis and Galinski, 1997; Kuhlmann and Bremer, 2002; Kuhlmann A.U. et al., 2008). The *ectD* gene can be part of the *ectABC* ectoine biosynthetic gene cluster, but it is often found elsewhere in the genome (Widderich et al., 2014, 2016). We found that the ectoine biosynthetic genes present in the genome sequence of *S. salinus* M19-40 are not genetically organized into the canonical *ectABC* operon. The *ectA* and *ectC* genes (SPISAL_06140 and SPISAL_06145) are found close together and their identification as part of the ectoine biosynthetic route seems straightforward. However, the unambiguous identification of the *ectB* gene turned out to be a bit more complicated. EctB is a L-2,4-diaminobutyrate-2-oxoglutarate transaminase (EC 2.6.1.76) and catalyzes the second step in ectoine biosynthesis (**Figure 2**; Ono et al., 1999). Transaminases participate in various biological transformations and we used therefore the amino acid sequence of the functionally characterized EctB enzyme from *H. elongata* (Ono et al., 1999; Schwibbert et al., 2011) as a template for a BLAST search of the *S. salinus* M19-40 genome. In this way, we found three EctB-related proteins: SPISAL_02400, SPISAL_06590, and SPISAL_07805 (León et al., 2013; Lopez-Perez et al., 2013). To distinguish the potential EctB enzyme from the two other transaminases, we used the orthology IDs from the KEGG database (Kanehisa et al., 2012). The SPISAL_07805 protein belongs to the orthology group K01845-hemL that comprises enzymes involved in heme biosynthesis. SPISAL_06590 belongs the orthology group K00821-argD comprises enzymes involved in arginine biosynthesis, and indeed the gene encoding SPISAL_06590 is flanked by a gene that is annotated in the *S. salinus* M19-40 genome sequence (León et al., 2013; Lopez-Perez et al., 2013) as an ornithine-carbamoyl-transferase (ArgF; SPISAL_06595). Bona fide EctB enzymes belong to the orthology group K00836-ectB but the SPISAL_02400 protein was not listed under this identification number in KEGG database. Instead, it was affiliated with two orthology groups: K00823-puuE (comprising 4-aminobutyrate aminotransferases) and K00821-argD. Since EctB is a L-2,4-diaminobutyrate-2-oxoglutarate transaminase (Ono et al., 1999), it seems to us that SPISAL_02400 participates in ectoine biosynthesis; however, we stress that this functional assignment requires further experimental scrutiny. We note that none of the *S. salinus* M19-40 SPISAL_02400, SPISAL_06590, and SPISAL_07805 proteins exhibits a high degree of amino acid sequence similarity to EctB proteins encoded by other ectoine biosynthetic gene clusters present in taxonomically relates species (**Figure 3**; see below).

Ectoine and 5-hydroxyectoine are not only effective osmostress protectants for many microorganisms, but they can also serve as nutrients. Genetic and biochemical analysis of *Sinorhizobium meliloti, R. pomeroyi* DSS-3, and *H. elongata* have provided insights into the 5-hydroxyectoine and ectoine catabolic route (Jebbar et al., 2005; Schwibbert et al., 2011; Schulz et al., 2017a,b). Only a minority of 5-hydroxyectoine/ectoine producers is also capable of 5-hydroxyectoine/ectoine catabolism (Schulz et al., 2017b). Inspection of the *S. salinus* M19-40 genome sequence showed that the core set of genes required for the use of ectoines as nutrients is missing.

### Non-canonical Genetic Organization of Ectoine Biosynthetic Genes in *S. salinus* M19-40 and Phylogenetically Related Species

Since the inspection of the *S. salinus* M19-40 genome sequence revealed an unusual genetic organization of the ectoine biosynthetic genes (**Figure 3**), we wondered whether this was an isolated incident or if this would occur more frequently. We therefore used the amino acid sequence of the *S. salinus* M19-40 ectoine synthase (EctC) in an initial BLAST search (Altschul et al., 1990) and analyzed the genetic organization of ectoine and 5-hydroxyectoine biosynthetic genes in the best fitting bacterial strains with known 16S rRNA sequences, resulting in 33 members of the *Gammaproteobacteria* and three of the *Deltaproteobacteria* with >78% EctC sequence identity. Since EctC is typically used as the diagnostic enzyme for the ectoine biosynthetic route (Louis and Galinski, 1997; Ono et al., 1999), it is conveniently employed in database searches to assess the presence of ectoine biosynthetic genes (Widderich et al., 2014, 2016). Strikingly, only nine of the 35 inspected genomes contained the canonical *ectABC* gene cluster (**Figure 3**). In most other cases, the genetic organization for the ectoine biosynthetic genes was scrambled. While the co-occurrence of the *ectA-ectC* genes in an apparent operon was shared by *S. salinus* M19-40 and *S. curvatus* UAH-SP71 (**Figure 3**), the only genome-sequenced *Spiribacter* species, other members of the family *Ectothiorhodospiraceae* contain a standard *ectABC* cluster with (*Arhodomonas*) or without (*Halorhodospira, Aquisalimonas, Nitrococcus*) an *ectD* gene, or a cluster with divergently oriented *ectC* and *ectAB* genes (*Alkalilimnicola*). Members of other gammaproteobacterial families contain standard *ect* gene clusters (*Halomonadaceae*), clusters with co-localized *ectA* and *ectB* genes, while *ectC* was located elsewhere in the genome sequences (most *Marinobacter* species/*Alteromonadaceae* or *Thioalkalimicrobium/Piscirickettsiaceae*), or do not show clustering of any *ect* genes (some *Marinobacter* strains/*Alteromonadaceae* or *Marinimicrobium*/Cellvibrionaceae). Moreover, some of the *Marinobacter* strains even contain two or three separate copies of *ectC* genes.

In our dataset, three members of the *Marinobacter* genus (*Marinobacter aquaeolei* VT8, *Marinobacter salsuginis* SD-14B, *Marinobacter* sp. CP1) possess two to three copies of the *ectC* gene (**Figure 3**). The amino acid sequences of the encoded proteins are closely related (Supplementary Figure 2A). Based on an alignment of these sequences we built a phylogenetic tree which suggest that two *ectC* copies within a given strain evolved from an ancestral gene duplication event and that the third *ectC* copy present in *M. aquaeolei* VT8 and *Marinobacter* sp. CP1 were derived from a more recent gene duplication (Supplementary Figure 2). Interestingly, in the vicinity of these latter additional *ectC* copies genes are found that encode a transposase.

Many of the studied gammaproteobacterial genome sequences also contained the gene (*ectD*) encoding the ectoine hydroxylase (Bursy et al., 2007; Höppner et al., 2014), but *ectD* was, with a single exception, never part of an *ect* gene cluster (**Figure 3**). Notably, both *S. salinus* M19-40 and *S. curvatus* UAH-SP71 lacked the *ectD* gene (**Figure 3**) while only two other members of the *Ectothiorhodospiraceae* present in our dataset contained *ectD* genes. The strains affiliated to the *Halomonadaceae* or *Alteromonadaceae* mostly contain *ectD* genes, and a considerable number of them contained even 2, or even 3, *ectD* copies (**Figure 3**). This phenomenon has been first functionally studied in *C. salexigens* by assessing the individual contributions of the two EctD-type proteins (referred to as EctD and EctE, respectively) to the overall 5-hydroxyectoine biosynthetic capacity of this bacterium. EctD contributed most to 5-hydroxyectoine production by *C. salexigens* since the expression of *ectE* was negligible under the studied growth conditions (Vargas et al., 2008). We compared the amino acid sequence of the ectoine hydroxylase from those five microorganisms that possess multiple *ectD* genes (**Figure 3**) and found that the amino acid sequence of each of these 11 EctD-type proteins is closely related to each other. Each of these proteins possesses the consensus sequence of *bona fide* ectoine hydroxylases (Supplementary Figure 3A), a stretch of 17 amino acids that is not only of structural importance for the formation of the cupin barrel (Reuter et al., 2010) but contains five residues involved in binding of the iron catalyst, the 2-oxoglutarate co-factor and the 5-hydroxyectoine reaction product (Höppner et al., 2014). A phylogenetic tree build using the amino acid sequence alignment suggests that each copy of the EctD protein present in a given strain has a different evolutionary history (Supplementary Figure 3B). We can, of course, not tell from our analysis in those microorganisms possessing multiple *ectD* genes, which copy of the EctD enzymes contributes mostly to 5-hydroxyectoine biosynthesis.

Neither the two sequenced *Spiribacter* species nor any other member of the *Ectothiorhodospiraceae* contained a gene for a specialized aspartokinase (Ask_Ect), an enzyme that is involved in producing the precursor for ectoine biosynthesis, beta-aspartate-semialdehyde (Reshetnikov et al., 2006; Stöveken et al., 2011). Nevertheless, this type gene (annotated as *ask*) is co-localized with *ectC* in 25 of the 35 studied genome sequences, which comprises all *Marinococcus* and *Marinimicrobium* strains (**Figure 3**). The biochemical properties of the Ask_Ect enzyme and its unusual type of feedback regulation by amino acids have been characterized for the corresponding protein from the ectoine/5-hydroxyectoine producer *Pseudomonas stutzeri* A1501, a plant-root-associated bacterium (Stöveken et al., 2011). Finally, all three strains of the *Deltaproteobacteria* present in our dataset (**Figure 3**) contain a conserved standard *ectABC* cluster with an added *ask* gene. However, they lack *ectD*, as expected from their physiology as strictly anaerobic sulfate reducers of two unrelated families since EctD is an oxygen-depending enzyme (Bursy et al., 2007; Höppner et al., 2014; Widderich et al., 2014). Most of these strains are of marine origin, either as obligate or facultative aerobic or as strictly anaerobic bacteria, which cover all marine lifestyles from pelagic to sediment habitats and may also occur together in tidal pools or saltern ponds. The presence of highly similar copies of apparent *ectABC-ask* operons in a few strains of sulfate-reducing *Deltaproteobacteria* affiliated to two unrelated families, and the lack of these genes in any of the genome-sequenced related species may be taken as indication for lateral gene transfer events.

In inspecting the genomic context of the ectoine biosynthetic genes, we noticed in many cases genes whose products are annotated as transporters. They belong to different transporter families and are mostly single-component systems (**Figure 3**). It remains to be seen, if these putative transporters are involved in the uptake of pre-formed ectoines from environmental sources (Kuhlmann et al., 2011), or if they participate in the recovery of newly synthesized ectoines that have been actively excreted (or leaked) across the cytoplasmic membrane (Grammann et al., 2002).

### Finely Tuned Synthesis of Ectoine by *S. salinus* M19-40 in Response to High Salinity

**FIGURE 4:**
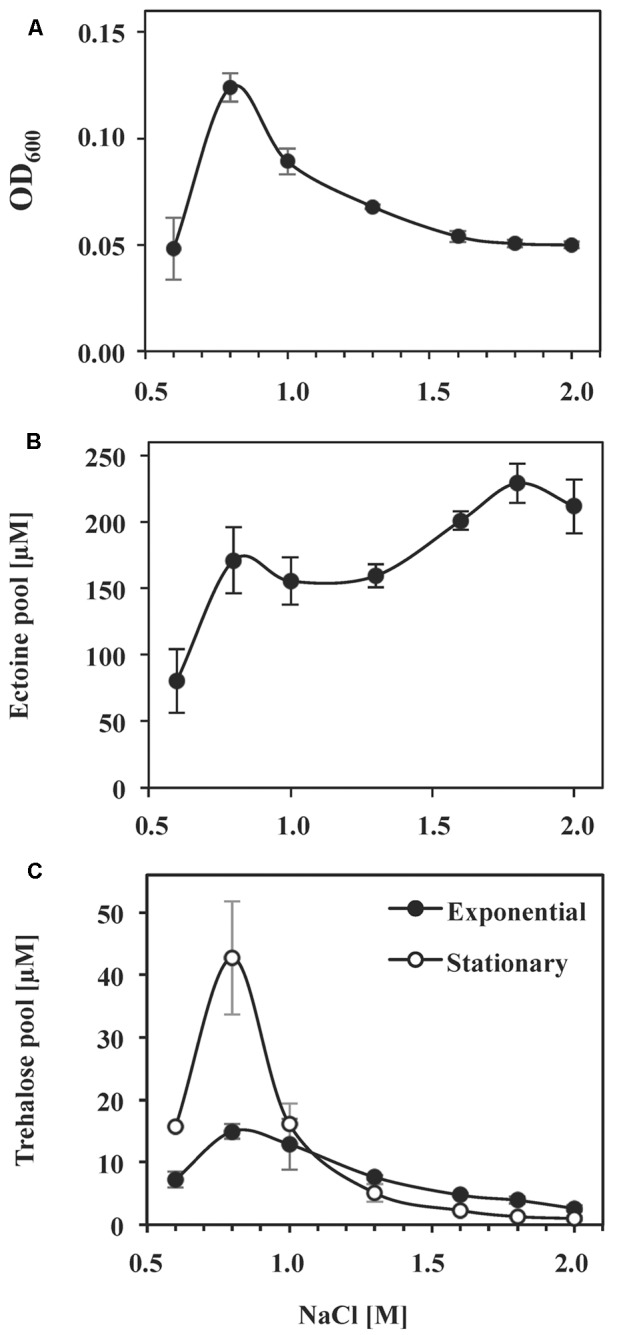
Effect of NaCl concentration and growth phase on the synthesis of ectoine and trehalose. *S. salinus* M19-40 cells were grown in SMM media of different NaCl concentrations. **(A)** Growth yields of the cultures (OD_600_) propagated were measured after 70 h of incubation. Intracellular ectoine **(B)** and trehalose **(C)** pools were analyzed in solvent extracts of cells taken from the cultures in their exponential growth phase after 70 h (closed circles) and in the stationary growth phase after 140 h (open circles) of incubation. The values given were calculated referring to an optical density (OD_600_) of 1 and are averages from three to six independent replicates. Standard deviations are given.

While the non-canonic arrangement of ectoine biosynthetic genes in a number of microorganisms has been noted before (Widderich et al., 2014, 2016), to the best of our knowledge, ectoine production has not been analyzed in any of them. To study ectoine synthesis by *S. salinus* M19-40 in response to salt stress in more detail, we quantified the intracellular ectoine pools of the cells by HPLC analysis. For these experiments, we grew *S. salinus* M19-40 in SMM medium possessing various salinities, extracted from the soluble solutes from the collected cells, and monitored ectoine production levels by HPLC analysis. These data demonstrate that ectoine biosynthesis in *S. salinus* M19-40 is responsive to the degree of salt stress imposed onto the cells (**Figures 4A,B**). The ectoine content of the cells increased about twofold from a basal level of 80 μM in cultures growing under sub-optimal saline conditions (SMM with 0.6 M NaCl) to 170 μM in cultures propagated at optimal salinity (SMM with 0.8 M NaCl) (**Figures 4A,B**). Although further increases in the salinity of the medium up to 1.3 M NaCl notably reduced growth yields, the ectoine pool did not increase concomitantly. Only severely growth restricting salt concentrations (1.6 M NaCl or higher) appear to trigger further moderate increases of the ectoine pools (e.g., 230 μM in cultures propagated in SMM with 1.8 M NaCl) (**Figures 4A,B**). Consistent with our finding that the *S. salinus* M19-40 genome sequence does not contain the gene for the ectoine hydroxylase (*ectD*), we did not detect any 5-hydroxyectoine in any of the cell extracts.

### Synthesis of Trehalose by *S. salinus* M19-40 in Response to High Salinity and Stationary Phase

Since mining of the *S. salinus* M19-40 genome sequence indicates that this bacterium possesses the *otsBA* genes, it should be able to synthesize the compatible solute trehalose (Avonce et al., 2006; **Figure 2**). We assessed the content of the same cell extracts for the presence of this sugar as used for ectoine quantitation. Trehalose was detected but was present in substantially reduced levels compared to those found for ectoine (**Figures 4B,C**). This indicates that trehalose contributes only moderately to balance the osmotic gradient across the cytoplasmic membrane of salt-challenged *S. salinus* M19-40 cells. Trehalose is not only used by microorganisms as an osmostress protectant but also serves as a cytoprotectant against other types of cellular and environmental stresses; e.g., challenges imposed by stationary growth phase (Hengge-Aronis et al., 1991; Strom and Kaasen, 1993). Accordingly, we found a strong increase in trehalose content of stationary phase cells of *S. salinus* M19-40 propagated under optimal salt concentrations (0.8 M NaCl), compared to exponentially growing cells (**Figure 4C**).

### Osmostress Protection of *S. salinus* M19-40 by an Exogenous Supply of Compatible Solutes

**FIGURE 5:**
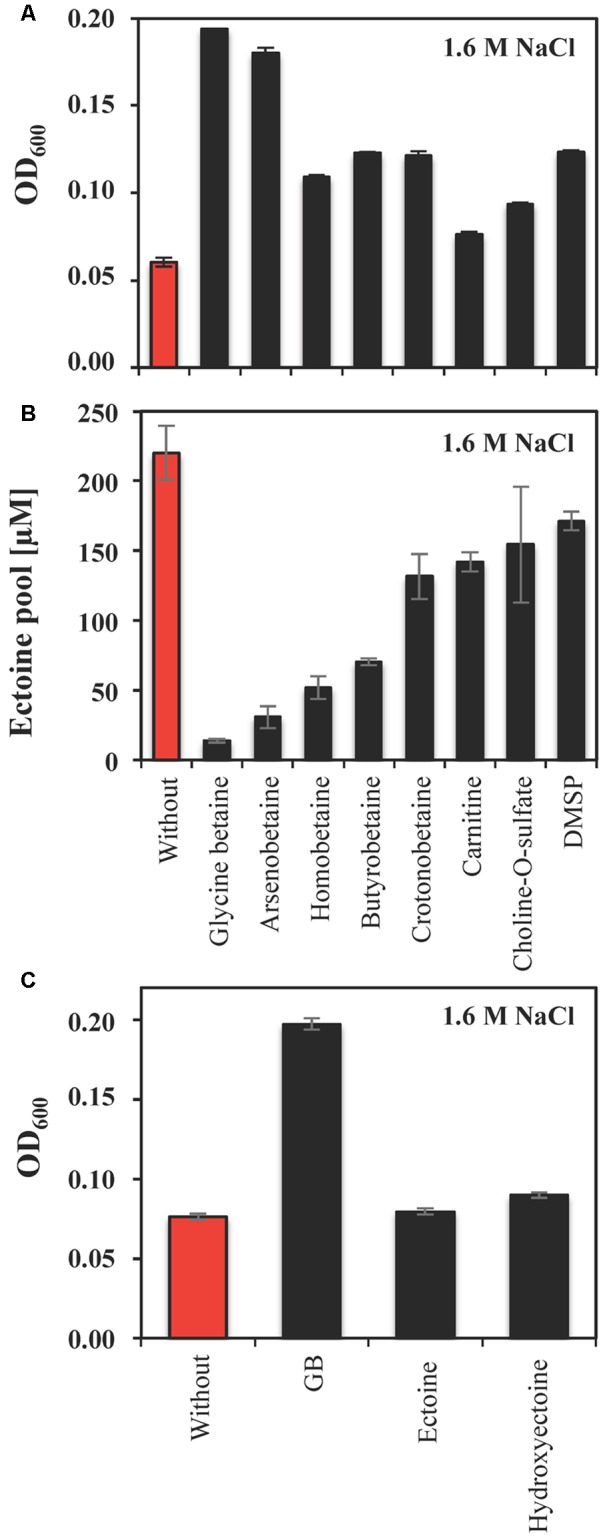
Osmostress protection by compatible solutes and corresponding ectoine pools of *S. salinus* M19-40 cells. **(A)** Cultures of *S. salinus* M19-40 were grown at 37°C in SMM containing 1.6 M NaCl. These cultures were propagated either in the absence (red bar) or in the presence of 1 mM of the indicated compatible solutes (black bars), and their growth yields were measured after 70 h. **(B)** Ethanolic extracts of cells taken from these cultures were analyzed for their ectoine content and were calculated referring to an optical density (OD_600_) of 1. **(C)** *S. salinus* M19-40 cells were grown at 37°C in SMM with 1.6 M NaCl and in the absence or the presence of 1 mM of glycine betaine, ectoine, or 5-hydroxyectoine, respectively. Growth yields were determined after 70 h. The values given are averages from three independent replicates. Standard deviations are given.

In most microorganisms, relief from high-salinity-imposed stress is accomplished through the import of compatible solutes via various types of high-affinity transport systems (Bremer and Krämer, 2000; Poolman et al., 2004; Krämer, 2010; Wood, 2011). Our *in silico* analysis of the *S. salinus* M19-40 genome sequence indicates the presence of several representatives of these systems (**Figure 2**). To experimentally assess whether *S. salinus* M19-40 is able to take advantage of compatible solutes provided in the growth medium, we tested the stress-protective properties of glycine betaine, arsenobetaine, homobetaine, γ-butyrobetaine, crotonobetaine, carnitine, choline-*O*-sulfate, and DMSP. For this set of experiments, we grew *S. salinus* M19-40 in SMM containing 1.6 M NaCl in the absence or the presence of 1 mM of the indicated compatible solutes and monitored the growth yield of these cultures. Under such high-salinity conditions (**Figure 1**), growth of *S. salinus* M19-40 was severely impaired (**Figure 5A**). Glycine betaine, and arsenobetaine provided strong osmostress protection while the other tested compatible solutes had more moderate stimulating effects for cell growth (**Figure 5A**). Since our *in silico* analysis of the *S. salinus* M19-40 genome indicates the presence of a TeaABC-related transporter (Grammann et al., 2002; **Figure 2**) we were particularly surprised that neither externally provided ectoine nor 5-hydroxyectoine afforded any notable osmostress protective effects (**Figure 5C**).

### Osmostress-Responsive Build-up of Glycine Betaine Pools

**FIGURE 6:**
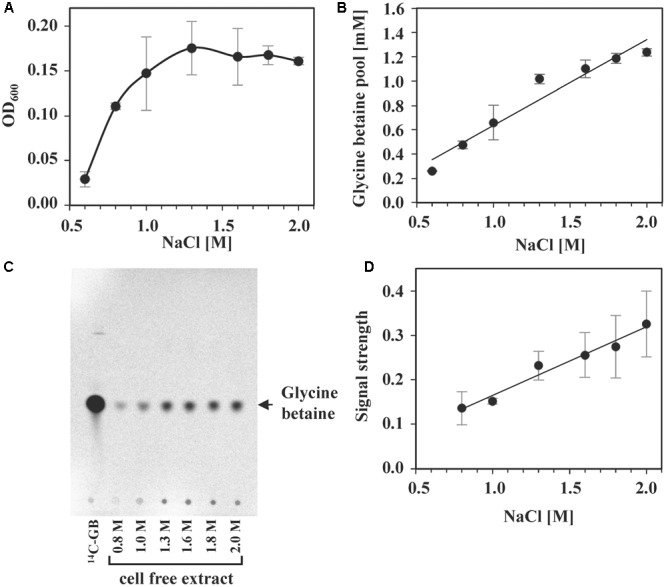
Intracellular glycine betaine accumulation in response to increasing NaCl concentrations. *S. salinus* M19-40 cultures were grown in three independent replicates, in SMM with increasing NaCl concentrations in the presence of 1 mM glycine betaine spiked with 0.64 μM [1-^14^C]glycine betaine. **(A)** Growth yields (OD_600_) of the cultures were measured after 70 h of incubation at 37°C. **(B)** Glycine betaine accumulated by the cells was determined by scintillation counting. The values given for each NaCl concentration were calculated referring to an optical density (OD_600_) of 1. **(C)** Cell-free extracts of the cells were separated by TLC, and radiolabeled compounds were visualized by autoradiography. [1-^14^C]glycine betaine was run on the thin-layer chromatography plate along the samples as a reference. Shown is a representative autoradiograph of one biological replicate. **(D)** The signals from three independent autoradiographs of TLC plates were quantified, normalized using the [1-^14^C]glycine betaine as internal concentration standard and then calculated referring to the OD_600_ of the cultures. All values given include averages and standard deviations of three independent replicates.

The presence of glycine betaine in the medium of high-salinity-challenged *S. salinus* M19-40 cultures grown in the presence of a broad range of salinities provided substantial osmostress protection, even for cells that were cultivated in SMM containing 2.0 M NaCl (**Figure 6A**). We found that radiolabeled glycine betaine was accumulated under steady-state osmotic stress conditions in a linear fashion in response to increases in the external salinity (**Figure 6B**). As proven by TLC, the amassed glycine betaine was not modified to any other compound by the *S. salinus* M19-40 cells (**Figures 6C,D**).

### Accumulation of Glycine Betaine Modulates the Ectoine Pools of the Cells

It is well established that the import and accumulation of osmostress protectants substantially suppresses the synthesis of the endogenously produced compatible solute by the bacterium under study (Hoffmann et al., 2013). This was also the case for ectoine biosynthesis by *S. salinus* M19-40 when the cells were cultivated in the presence of various compatible solutes. The effectiveness of this suppression varied (**Figures 5A,B**), with glycine betaine and arsenobetaine having the greatest influence on the cellular ectoine pool (**Figure 5B**). Moreover, the relative osmotic protection and suppression of ectoine biosynthesis appear to deviate for the various tested effectors, suggesting independent effects causing these observations.

**FIGURE 7:**
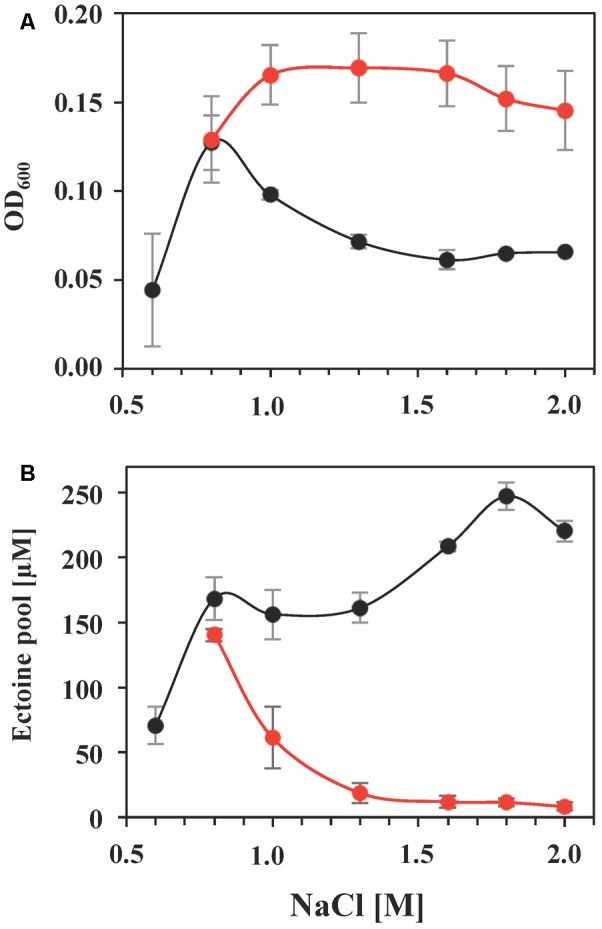
Growth protection by glycine betaine and its influence on the intracellular ectoine pool. *S. salinus* M19-40 cultures were grown in SMM with increasing NaCl concentrations either in the absence (black circles) or the presence (red circles) of 1 mM glycine betaine. **(A)** Growth yields (OD_600_) of the cultures measured after 70 h of incubation at 37°C. **(B)** Ectoine pools were determined in ethanolic extracts of cells harvested from the cultures that were grown to exponential growth phase after 70 h of incubation. The values given were calculated referring to an optical density (OD_600_) of 1 and are averages from three independent replicates. Standard deviations are given.

Using glycine betaine as an example, we investigated this effect in greater detail in cultures grown in the presence of a broad range of salinities. Glycine betaine provided a substantial degree of osmostress protection when the salinity of the medium exceeded that for optimal growth (**Figure 7A**). Concomitantly, the intracellular ectoine pool was successively reduced to a basal level, a 17-fold drop when the ectoine pools of *S. salinus* M19-40 cells grown under optimal salt concentrations (0.8 M NaCl) were compared with those of severely salt stressed cells (grown in the presence of 2 M NaCl) (**Figure 7B**).

## Discussion

High salinity is a key determinant for the growth of *S. salinus* M19-40 in the saltern ponds from which it was originally isolated (León et al., 2013, 2014; Lopez-Perez et al., 2013). Such a challenging habitat (Ventosa et al., 2014, 2015) requires active measures by this moderate halophile to counteract the outflow of water from the cells and to optimize the solvent properties of the cytoplasm for biochemical reactions and the functionality of cell components (Bremer and Krämer, 2000; Bolen and Baskakov, 2001; Wood, 2011). Analysis of the *in silico*-derived proteome and the calculation of isoelectric points of all predicted proteins of *S. salinus* M19-40 suggested that this bacterium uses the *salt-out* strategy (León et al., 2013; Lopez-Perez et al., 2013). The data presented here support this conclusion. Our experimental and *in silico*-derived findings highlight the ability of *S. salinus* M19-40 to cope with persistent high-salinity ecosystems through transport-mediated control over its cytoplasmic potassium and sodium pools, the synthesis and accumulation of the effective compatible solute ectoine, and to a minor extent through the synthesis of trehalose. *S. salinus* M19-40 also can derive a considerable level of osmostress protection through the import of glycine betaine and its arsenic homolog arsenobetaine. Glycine betaine uptake and trehalose/2-sulfotrehalose biosynthesis are also widely spread mechanisms for osmoadaptation in *Halobacteriales*, halophilic archaea that often dominate hypersaline habitats (Youssef et al., 2014).

The ability to synthesize the osmostress protectant ectoine is found commonly in nature, including in marine microorganisms, those living in high-salinity ecosystems, and in habitats in which the osmolarity/salinity fluctuates (Pastor et al., 2010; Widderich et al., 2014, 2016). The genetic organization of the ectoine biosynthetic genes from *S. salinus* M19-40 deviate from their canonical arrangement as an *ectABC* operon (Louis and Galinski, 1997; Kuhlmann A.U. et al., 2008; Pastor et al., 2010; Schwibbert et al., 2011; Widderich et al., 2014, 2016) in that at least one gene is separated from the other two (**Figure 3**). Our database searches revealed that other non-canonical gene arrangements of the *ect* biosynthetic genes can also be found in many groups of marine bacteria containing *ectC* gene products closely related to that of *S. salinus* M19-40 (**Figure 3**). To the best of our knowledge, the data reported here address for the first time ectoine biosynthesis in a bacterium with such a scrambled arrangement of ectoine biosynthetic genes. Regardless of the unusual genetic organization of the ectoine biosynthetic genes, *S. salinus* M19-40 controls the ectoine biosynthetic pathway in a highly coordinated manner and produces ectoine in a fashion that is linked to the level of salinity prevalent in the environment (**Figure 4B**). Currently, there is no genetic system available for *S. salinus* M19-40 that would allow the construction of a mutant defective in ectoine biosynthesis to assess the physiological consequence of an *ect* gene knock-out. However, such a genetic analysis has been carried out in *C. salexigens*, a phylogenetically related ectoine producer (Vargas et al., 2008; **Figure 3**). As expected, the disruption of the ectoine biosynthetic genes causes osmotic sensitivity (Canovas et al., 1997), and we presume that this would also be the case for *S. salinus* M19-40.

*Spiribacter salinus* M19-40 is an abundant microorganism in saltern ponds with intermediate salinities (León et al., 2013, 2014; Lopez-Perez et al., 2013; Ventosa et al., 2014). As a consequence, cell lysis of ectoine-loaded *S. salinus* M19-40 cells will contribute, in all likelihood, significantly to the environmental ectoine pool that in turn can then be exploited by other inhabitants of this ecosystem, either as an effective osmostress protectant (Pastor et al., 2010) or by using this nitrogen-rich compound as a valuable nutrient (Schwibbert et al., 2011; Schulz et al., 2017a,b). In contrast to *H. elongata* (Schwibbert et al., 2011) and *C. salexigens* (Vargas et al., 2006), bacteria that are phylogenetically affiliated to the *Halomonadaceae*, the genome of *S. salinus* M19-40 (León et al., 2013; Lopez-Perez et al., 2013) lacks the core genes required for ectoine catabolism (Schwibbert et al., 2011; Schulz et al., 2017a,b). These genes are also not present in the genome sequence of any other bacteria that are phylogenetically related to *S. salinus* M19-40 whose genome sequence we inspected in this study (**Figure 3**).

The data presented here report for the first time the synthesis of trehalose in any member of the *Spiribacter* genus (**Figure 4C**). The low pool size of this compatible solute, however, suggests that it probably plays only a minor role in osmostress adjustment for *S. salinus* M19-40. There is, however, an up-regulation of the trehalose pool in stationary phase cells suggesting that its production might be primarily associated with the amelioration of types of stresses other than high osmolarity/salinity. For instance, enhanced trehalose production has also been observed when *E. coli* cells enter stationary phase (Giaever et al., 1988; Hengge-Aronis et al., 1991), a growth phase that imposes many constraints onto the bacterial cell (Hengge-Aronis, 2002).

We found that *S. salinus* M19-40 can exploit exogenously provided compatible solutes for osmostress protection, thereby broadening the range of salinities where optimal growth can occur (**Figures 5A, 7A**). Glycine betaine, probably the most widely used compatible solute on Earth (Yancey, 2005), and its arsenic homolog (Popowich et al., 2016; Hoffmann et al., 2017), provided the most effective degree of osmostress protection. Our genome mining data and modeling studies of predicted substrate-binding proteins (ProX and OpuAC; Supplementary Figures 4, 5; Schiefner et al., 2004; Horn et al., 2006) of the *S. salinus* M19-40 ProU- and OpuA-type ABC transporters suggest that these osmolyte importers are involved in scavenging glycine betaine (**Figure 2**). Arsenobetaine is widely found in marine ecosystems (Popowich et al., 2016) and recent studies with *B. subtilis* as the model system suggest that in essence any glycine betaine transporter should also be able to import the arsenic homolog (Hoffmann et al., 2017). Indeed, the ABC transporter OpuA and the BCCT-type transporter OpuD from *B. subtilis* can scavenge arsenobetaine effectively from the growth medium (Hoffmann et al., 2017). The genes for two BCCT-type transporters (OpuD-1; OpuD-2) are found in the genome sequence of *S. salinus* M19-40 (León et al., 2013; Lopez-Perez et al., 2013). Proteins belonging to the BCCT-family are closely related in their amino acid sequence and topological organization within the membrane, but their substrate specificity cannot be predicted with confidence by simply inspecting their amino acid sequence (Ziegler et al., 2010). The osmotically inducible TRAP-type ectoine/5-hydroxyectoine transporter from *H. elongata* serves for the import of these compatible solutes when they are used as osmostress protectants (Grammann et al., 2002). Amino acid sequence comparison and modeling studies (Supplementary Figure 6) based upon the crystal structure TeaA::ectoine complex of the periplasmic substrate binding protein (Kuhlmann S.I. et al., 2008) strongly suggest that the predicted TeaABC TRAP-transporter from *S. salinus* M19-40 (**Figure 2**) should be able to import ectoines. However, contrary to this expectation, exogenously provided ectoines did not serve as osmostress protectants for *S. salinus* M19-40 (**Figure 5C**). This is a highly surprising finding and we have currently no reasonable explanation for this observation. In any event, follow-up studies are required to define the precise substrate spectrum of the OpuA, ProU, OpuD-1, OpuD-2, and TeaA transporters (**Figure 2**), and such studies will require the construction of a set of isogenic gene disruption mutations, as, for instance, has been intensively used to define the substrate profile of the osmotically controlled five Opu osmolyte transporters operating in *B. subtilis* (Hoffmann and Bremer, 2016, 2017).

The import of glycine betaine strongly down-regulated the steady-state ectoine pool of *S. salinus* M19-40 (**Figure 7**), indicating that this bacterium prefers glycine betaine as an osmostress protectant. Either because of the superior physico-chemical properties of this chemical chaperon (Zaccai et al., 2016; Stadmiller et al., 2017) or to save precursors and biosynthetic resources used for ectoine production (Pastor et al., 2010; Oren, 2011; Widderich et al., 2014). It is known from other studies, that the down-regulation of the ectoine pool in response to the import of glycine betaine or other compatible solutes is primarily the consequence of the down-regulation of the transcription of the ectoine biosynthetic genes (Bestvater et al., 2008; Czech et al., 2018).

In summary, our data suggest that ectoine biosynthesis and glycine betaine/arsenobetaine uptake are key features for the physiological adjustment process of *S. salinus* M19-40 to sustained but moderately high saline habitats. Nevertheless, the growth profile of *S. salinus* M19-40 suggests that it is optimally adapted to a rather narrow range of salinities (**Figure 1**), an observation that is consistent with cultivation experiments and metagenomic data of the habitats from which different members of this genus were isolated (León et al., 2013, 2014, 2015, 2016, 2017; Lopez-Perez et al., 2013). However, its ability to scavenge compatible solutes, in particular glycine betaine and arsenobetaine, from environmental sources will allow *S. salinus* M19-40 to proliferate under otherwise growth-restricting salinities.

## Author Contributions

AV and EB conceived and supervised this study. ML, TH, and CS-P conducted the experiments and analyzed their results. JH helped with the interpretation of genomic data. TH, JH, AV, and EB wrote the paper.

## Conflict of Interest Statement

The authors declare that the research was conducted in the absence of any commercial or financial relationships that could be construed as a potential conflict of interest.

## Footnotes

1 http://jgi.doe.gov/data-and-tools/img/

2 https://www.arb-silva.de/

3 http://evolution.genetics.washington.edu/phylip/doc/

4 http://swissmodel.expasy.org/

5 http://www.pymol.org
