# Supplementary material for: Compatible Solute Synthesis and Import by the Moderate Halophile Spiribacter salinus: Physiology and Genomics
Source: Front Microbiol. 2018 Feb 15;9:108. doi: 10.3389/fmicb.2018.00108 (PMC5818414; doi:10.3389/fmicb.2018.00108)
Supplement: Supplementary file 1 [file Presentation_1.PDF]

Supplementary Material

Compatible Solute Synthesis and Import in the Moderate Halophile *Spiribacter salinus*: Physiology and Genomics

María José León[1¶], Tamara Hoffmann[2¶], Cristina Sánchez-Porro[1], Johann Heider[2,3], Antonio Ventosa[1*], and Erhard Bremer[2,3*]

[1]Department of Microbiology and Parasitology, Faculty of Pharmacy, University of Sevilla, Sevilla, Spain
[2]Laboratory for Microbiology, Department of Biology, Philipps-University Marburg, Marburg, Germany
[3]LOEWE-Center for Synthetic Microbiology, Philipps-University Marburg, Marburg, Germany

[¶]These authors contributed equally

For correspondence during the reviewing and editorial process please contact:
Dr. Erhard Bremer, Philipps-University Marburg, Dept. of Biology, Laboratory for Microbiology, Karl-von-Frisch-Str. 8, D-35032 Marburg, Germany. Phone: (+49)-6421-2821529. Fax: (+49)-6421-2828979. E-Mail: bremer@staff.uni-marburg.de

*Correspondence:
Antonio Ventosa: ventosa@us.es
Erhard Bremer: bremer@staff.uni-marburg.de

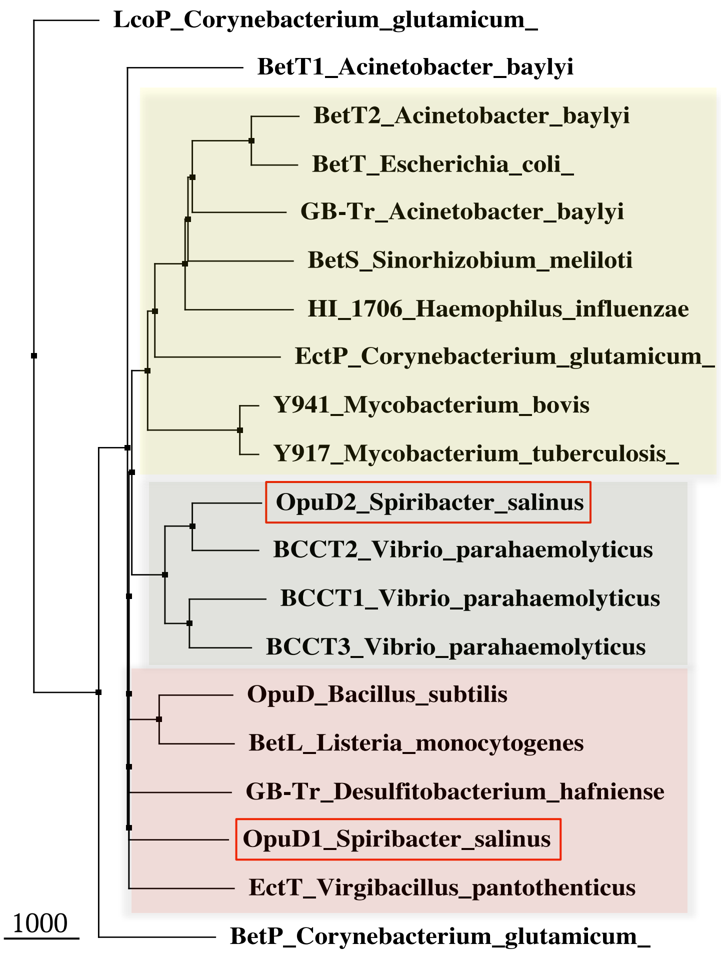

Supplementary Figure 1. Phylogenetic tree of protein sequences identified via a BLAST analysis using the OpuD1 (SPISAL_05155) and OpuD2 (SPISAL_05630) amino acid sequences of *S. salinus* M19-40 as the search template against the UniProt Knowledgebase (http://www.uniprot.org/). The indicated phylogenetic distances are based on an alignment of the amino acid sequences that was created with the CLUSTAL OMEGA algorithm (Sievers et al., 2011).

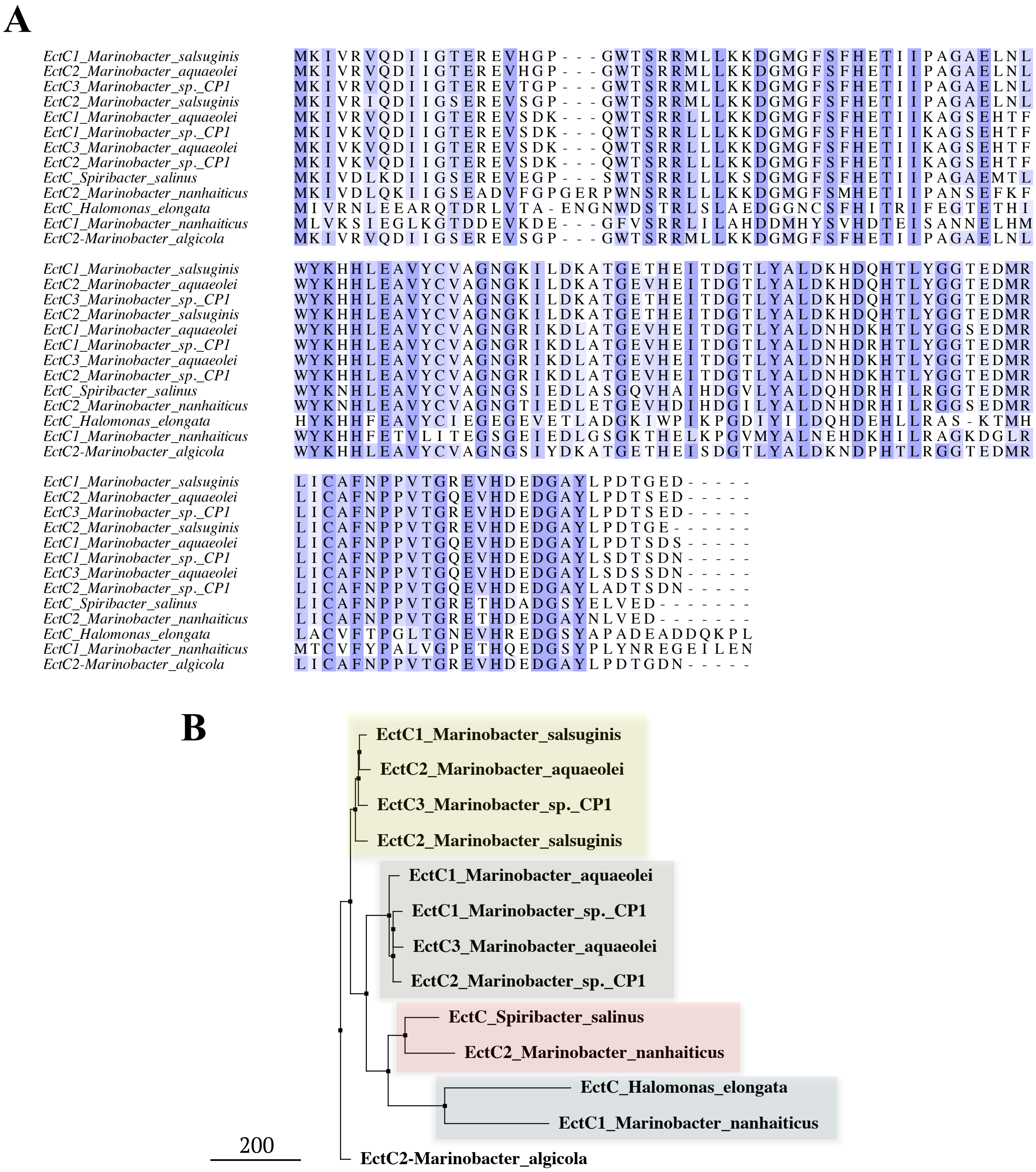

Supplementary Figure 2. Sequence comparison of EctC proteins that exist as homologues in the same organism. The amino acid sequences of three EctC homologues in *Marinobacter aquaeolei* VT8 (EctC1 [Maqu_0444], EctC2 [Maqu_0079], EctC3 [Maqu_0616]), two EctC homologues in *Marinobacter salsuginis* SD-14B (EctC1 [Msal_04565], EctC2 [Msal_04384]), three EctC homologues in *Marinobacter sp*. CP1 (EctC1 [Ga0098240_112317], EctC2 [Ga0098240_112233], EctC3 [Ga0098240_11115]) and two EctC homologues in *Marinobacter nanhaiticus* (EctC1 [J057_15490] and EctC2 [J057_07276]) were compared to the EctC proteins of *S. salinus* M19-40 [SPISAL_06145] and *Halomonas elongata* [HELO_2590]. (A) EctC amino acid sequences were aligned using the CLUSTAL OMEGA algorithm (Sievers et al., 2011). (B) The phylogenetic tree was built on the basis of the EctC amino acid alignments.

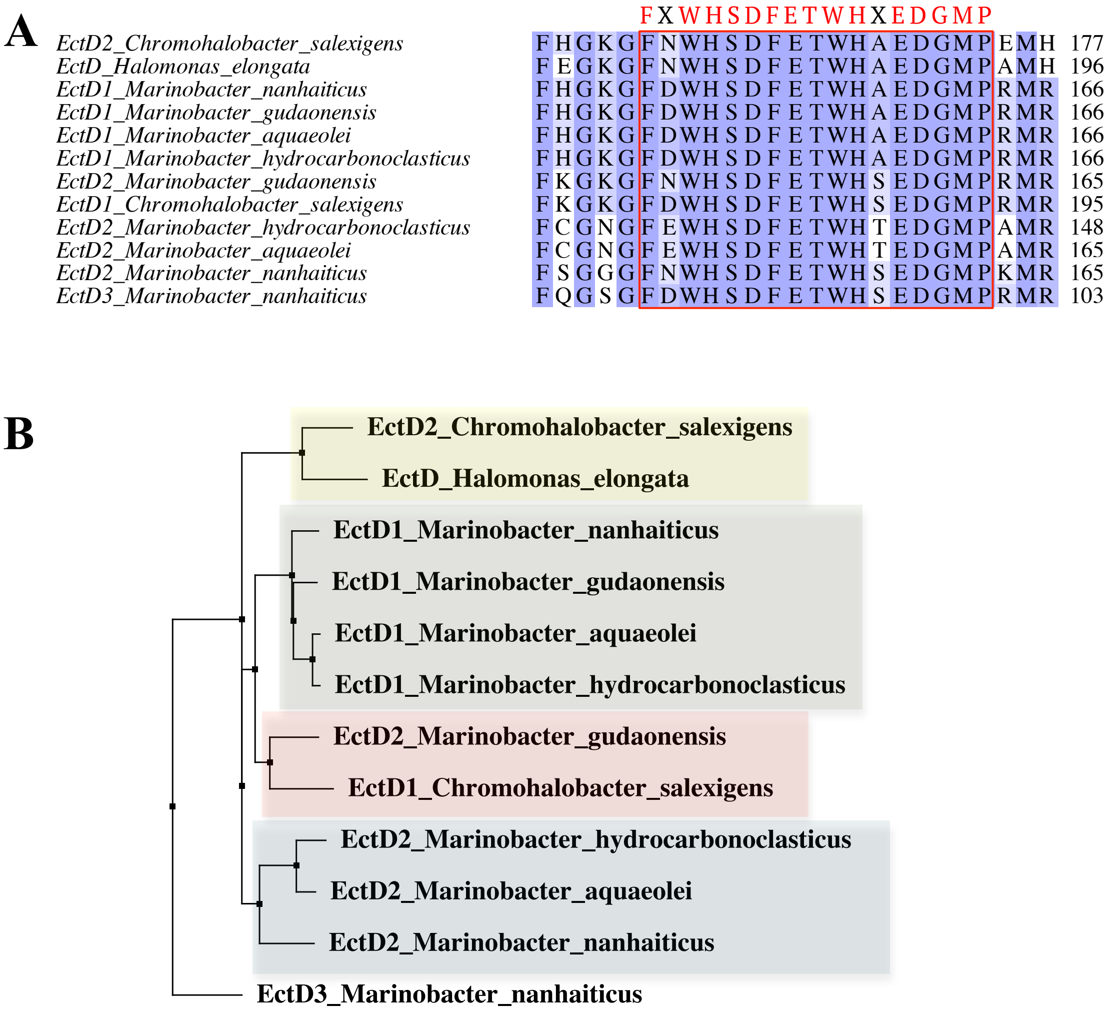

Supplementary Figure 3. Sequence comparison of EctD proteins that exist as homologues in one microorganism. The amino acid sequences from two EctD proteins from *Chromohalobacter salexigens* (EctD1 [Csal_3003] and EctD2 [Csal_0542], three EctD proteins from *Marinobacter nanhaiticus* D15-8W (EctD1 [J057_08271], EctD2 [J057_16265] and EctD3 [J057_19620]), two EctD proteins from *Marinobacter gudaonensis* CGMCC 1.6294 (EctD1 [Ga0070159_2058] and EctD2 [Ga0070159_1429]), two EctD proteins from *Marinobacter aquaeolei* VT8 (EctD1 [Maqu_3892] and EctD2 [Maqu_1849]) and two EctD proteins from *Marinobacter hydrocarbonoclasticus* ATCC 49840 (EctD1 [MARHY3849] and EctD2 [MARHY1452]) were compared with the EctD protein of *Halomonas elongata* [HELO_4008]. (A) The EctD amino acid sequences were aligned using the CLUSTAL OMEGA algorithm (Sievers et al., 2011). The 17 amino acids consensus sequence of ectoine hydroxylases (Höppner et al., 2014) is given in red . (B) The phylogenetic tree was built on the basis of the amino acid alignment of the various EctD proteins.

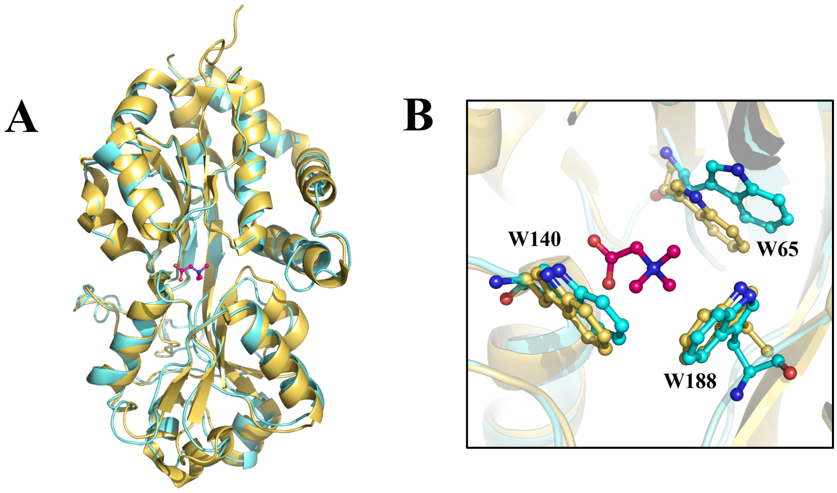

Supplementary Figure 4 *in silico* model of the ProX homolog of the *S. salinus* M19-40 SPISAL_05285 ligand-binding protein in complex with glycine betaine. The *Escherichia coli* ProU system is a ABC-type transporter and consists of the ProVWX subunits; ProX is the periplasmic substrate-binding protein of the ProU transporter. The ProX::glycine betaine complex of *E. coli* was used as the template for modeling and its crystal structure was taken from the PDB database (PDB 1R9L) (Schiefner et al., 2006). (**A**) Overall structure of the *S. salinus* ProX model (cyan) [QMEAN4: -9.58 (Arnold et al., 2006)] overlaid with the crystal structure of the ProX::glycine betaine complex from *E. coli* (yellow). (**B**) The ligand-binding site of the *E. coli* ProX protein with the three Trp (W) residues forming the aromatic cage for the coordination of the trimethylammonium head group of glycine betaine is shown (Schiefner et al., 2006). Within this hydrophobic pocket, the positively charged trimethlyammonium head-group of glycine betaine is coordinated via cation-π interactions and the carboxylate of the glycine betaine ligand protrudes out of the aromatic cage.

A B

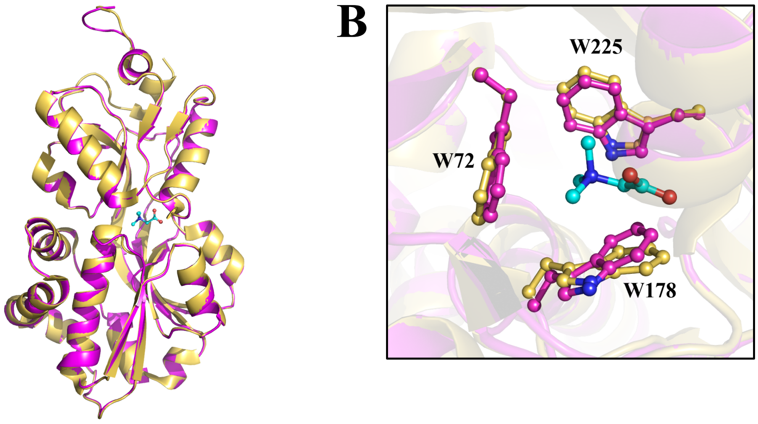

Supplementary Figure 5 *in silico* model of the OpuAC homolog of *S. salinus* M19-40 (SPISAL_06004) in complex with glycine betaine. The *Bacillus subtilis* OpuA system is a ABC-type transporter and consists of the OpuAA-OpuAB-OpuAC subunits; OpuAC is the extracellular substrate-binding protein of the OpuA transporter and is tethered to the outer-face of the cytoplasmic membrane via a lipid modification of the N-treminal Cys residue. The OpuAC::glycine betaine complex of *B. subtilis* was used as the template for modeling; the crystal structure data of the OpuAC::glycine betaine complex were taken from the PDB database (PDB 2B4L) (Horn et al., 2006). (**A**) Overall structure of the *S. salinus* M19-40 OpuAC model (pink) [QMEAN4: -5.5 (Arnold et al., 2006)] overlaid with the protein structure of the OpuAC::glycine betaine complex from *B. subtilis* (yellow)) (Horn et al., 2006). (**B**) The ligand-binding site of OpuAC with its aromatic cage formed by three Trp (W) residues is shown. Within this hydrophobic pocket, the positively charged trimethlyammonium head-group of glycine betaine is coordinated via cation-π interactions and the carboxylate of the glycine betaine ligand protrudes out of the aromatic cage.

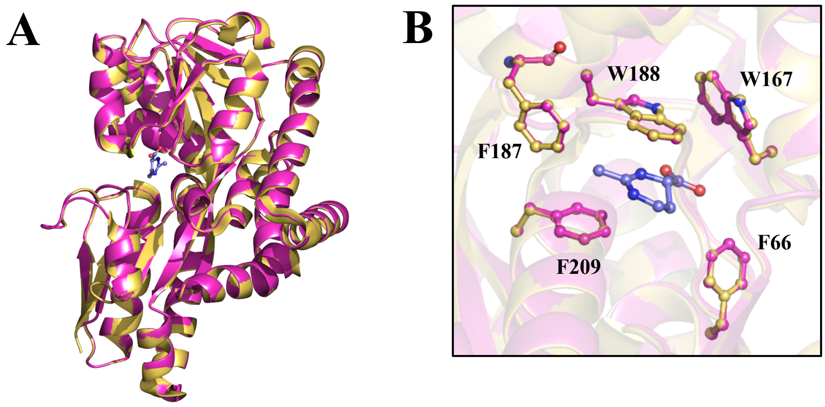

Supplementary Figure 6 *in silico* model of the TeaA homolog of the *S. salinus* M19-40 SPISAL_01895 ligand-binding protein in complex with ectoine. TeaABC system from *Halomonas elongata* is a TRAP-type transport system. The crystal structure of the TeaA::ectoine complex of *H elongata* was used as the template for modeling and its structural data were taken from the PDB database (PDB 2VPN) (Kuhlmann et al., 2008). (**A**) Overall structure of the *S. salinus* M19-40 TeaA model (magenta) [QMEAN4: -6.38 (Arnold et al., 2006)] overlaid with the crystal structure of the TeaA::ectoine complex from *H. elongata* (yellow) (Kuhlmann et al., 2008). (**B**) The active site of the TeaA ligand binding protein is overlaid with the model for the *S. salinus* M19-40 SPISAL_01895 protein.

Supplementary Table 1. Mining of the *S. salinus* M19-40 genome sequence

Name	Locus tag	Annotation[a)]	Search Template	Organism	Accession No.[b)]	e Value
**potassium uptake**						
TrkA	SPISAL_00310	uptake protein-TrkA	TrkA	*Escherichia. coli*	CAA54371.1	0E+00
TrkH	SPISAL_00315	potassium uptake protein TrkH	TrkG	*E. coli*	BAA14960.1	5E-105
			TrkH	*E. coli*	BAE77454.1	3E-158
TrkA	SPISAL_06575	TrkA	TrkA	*E. coli*	CAA71360.1	2E+00
TrkG	SPISAL_06570	Trk-type K+ transport system, membrane component	TrkG	*E. coli*	BAA14960.1	2E-26
			TrkH	*E. coli*	BAE77454.1	1E-25
**potassium efflux**						
	SPISAL_01180	potassium efflux system protein	KefC	*E. coli*	CTX42374.1	7E-69
**sodium efflux**						
MrpA/D	SPISAL_06665	monovalent cation/H+ antiporter subunit D	MrpA	*Bacillus subtilis*	AGG62567.1	1E-40
			MrpD	*B. subtilis*	AGG62570.1	3E-38
MrpA/D	SPISAL_06670	NADH dehydrogenase (quinone)	MrpA	*Bacillus subtilis*	AGG62567.1	2E-41
			MrpD	*B. subtilis*	AGG62570.1	1E-19
MrpA/D	SPISAL_06680	monovalent cation/H+ antiporter subunit D	MrpA	*Bacillus subtilis*	AGG62567.1	3E-29
			MrpD	*B. subtilis*	AGG62570.1	1E-23
MrpB	SPISAL_06650	monovalent cation/H+ antiporter subunit B				
MrpB	SPISAL_06655	monovalent cation/H+ antiporter subunit B				
MrpC	SPISAL_06660	NADH-ubiquinone oxidoreductase chain 4L	MrpC	*B. subtilis*	AGG62569.1	2E-08
MrpE	SPISAL_06635	cation antiporter	MrpE	*B. subtilis*	AGG62571.1	1E-07
MrpF	SPISAL_06640	multiple resistance and pH regulation protein F				
MrpG	SPISAL_06645	monovalent cation/proton antiporter subunit MnhG/PhaG				
**compatible solute uptake systems**						
OpuAC	SPISAL_06400	glycine betaine/proline transport system substrate-binding protein	OpuAC	*B. subtilis*	NP_388182.1	9E-36
OpuAB	SPISAL_06405	proline/glycine betaine ABC transporter permease	OpuAB	*B. subtilis*	NP_388181.1	7E-66
OpuAA	SPISAL_06410	glycine betaine/L-proline ABC transporter ATPase	+ neighbor			
ProW	SPISAL_05290	glycine betaine/proline ABC transporter permease	OpuAB	*B. subtilis*	NP_388181.1	1E-38
ProV	SPISAL_05295	glycine betaine/proline ABC transporter ATP-binding protein	ProW	*E. coli*	BAA16543.1	1E-44
ProX	SPISAL_05285	glycine betaine ABC transporter substrate-binding protein	+ neighbor			

a) Original gene product name (IMG product name).
b) Sequence accession.version number according to the NCBI protein database.

Supplementary Table 1. Mining of the *S. salinus* M19-40 genomes sequence (*continue*)

Name	Locus tag	Annotation[a)]	Search Template	Organism	Accession No.[b)]	e Value
OpuD1	SPISAL_05155	high-affinity choline uptake protein	OpuD	*B. subtilis*	NP_390885.1	7E-140
			BetH	*Halobacillus trueperi*	AAS05826.1	1E-131
			EctT	*V. pantothenticus*	AAL16076.1	2E-107
OpuD2	SPISAL_05630	choline/carnitine/betaine transporter	OpuD	*B. subtilis*	NP_390885.1	2E-108
			BetH	*H. trueperi*	AAS05826.1	1E-90
			EctT	*V. pantothenticus*	AAL16076.1	8E-71
TeaA	SPISAL_01895	TRAP transporter substrate-binding protein TeaA	TeaA	*Halomonas elongata*	CBV44158.1	0E+00
			UehA	*Ruegeria pomeroyi*	Q5LUA7.1	4E-129
TeaB	SPISAL_01890	TRAP transporter small transmembrane protein TeaB	UehB	*R. pomeroyi*	Q5LUA8.1	2E-76
TeaC	SPISAL_01885	transporter subunit; dicarboxylate transporter (IMG: DctM subunit)	UehC	*R. pomeroyi*	Q5LUA9.1	0E+00
Usp	SPISAL_01880	regulatory protein TeaD (IMG UspA-family)				
	SPISAL_04635	TRAP dicarboxylate transporter- DctP subunit	TeaA	*H. elongata*	CBV44158.1	2E+00
	SPISAL_04635	TRAP dicarboxylate transporter- DctP subunit	UehA	*R. pomeroyi*	Q5LUA7.1	8E-21
	SPISAL_04640	tripartite ATP-independent periplasmic transporter DctQ	UehB	*R. pomeroyi*	Q5LUA8.1	1E+00
	SPISAL_04645	TRAP dicarboxylate transporter subunit DctM	UehC	*R. pomeroyi*	Q5LUA9.1	7E-22
**ectoine synthesis**						
EctA	SPISAL_06140	diaminobutyrate acetyltransferase	EctA	*V. pantothenticus*	AAS93806.1	2E-39
EctB	SPISAL_02400	4-aminobutyrate aminotransferase	EctB	*V. pantothenticus*	AAS93807.1	8E-47
EctC	SPISAL_06145	ectoine synthase	EctC	*V. pantothenticus*	AAS93808.1	3E-46
**trehalose synthesis**						
OtsB	SPISAL_07860	HAD family hydrolase	OtsB	*E. coli*	BAA15718.1	9E-54
	SPISAL_07865	glycoside hydrolase 15-like protein				
OtsA	SPISAL_07870	alpha,alpha-trehalose-phosphate synthase	OtsA	*E. coli*	BAA15717.2	9E-123
**glucose uptake**						
Glk	SPISAL_03590	glucokinase	Glk	*E. coli*	KOZ53643.1	2E-51
**mechanosensitive channels**						
MscS	SPISAL_03885	mechanosensitive ion channel MscS	MscS (YkuT)	*B. subtilis*	NP_389304.2	2E+00
	SPISAL_03885	mechanosensitive ion channel MscS	MscS	*E. coli*	BAI32234.1	3E+00

a) Original gene product name (IMG product name).

b) Sequence accession.version number according to the NCBI protein database.

Supplementary References

Arnold, K., Bordoli, L., Kopp, J., and Schwede, T. (2006). The SWISS-MODEL workspace: a web-based environment for protein structure homology modelling. *Bioinformatics* 22**,** 195-201. doi: 10.1093/bioinformatics/bti770.

Höppner, A., Widderich, N., Lenders, M., Bremer, E., and Smits, S.H.J. (2014). Crystal structure of the ectoine hydroxylase, a snapshot of the active site. *J Biol Chem* 289**,** 29570-29583. doi: Doi 10.1074/Jbc.M114.576769.

Horn, C., Sohn-Bösser, L., Breed, J., Welte, W., Schmitt, L., and Bremer, E. (2006). Molecular determinants for substrate specificity of the ligand-binding protein OpuAC from *Bacillus subtilis* for the compatible solutes glycine betaine and proline betaine. *J. Biol. Chem.* 357, 592-606.

Kuhlmann, S.I., Terwisscha van Scheltinga, A.C., Bienert, R., Kunte, H.J., and Ziegler, C. (2008). 1.55 A structure of the ectoine binding protein TeaA of the osmoregulated Trap-Transporter TeaABC from *Halomonas elongata*. *Biochemistry* 47, 9475-9485. doi: 10.1021/bi8006719

Schiefner, A., Breed, J., Bösser, L., Kneip, S., Gade, J., Holtmann, G., et al. (2004). Cation-pi Interactions as determinants for binding of the compatible solutes glycine betaine and proline betaine by the periplasmic ligand-binding protein ProX from *Escherichia coli*. *J. Mol. Biol.* 279, 5588-5596.

Sievers F, Wilm A, Dineen DG, Gibson TJ, Karplus K, Li W, Lopez R, McWilliam H, Remmert M, Söding J, Thompson JD, Higgins DG (2011). Fast, scalable generation of high-quality protein multiple sequence alignments using Clustal Omega. Molecular Systems Biology 7:539 doi:10.1038/msb.2011.75
